# Prevalence, Comorbidity, and Sociodemographic Correlates of Psychiatric Disorders Reported in the All of Us Research Program

**DOI:** 10.1001/jamapsychiatry.2022.0685

**Published:** 2022-04-20

**Authors:** Peter B. Barr, Tim B. Bigdeli, Jacquelyn L. Meyers

**Affiliations:** 1 Department of Psychiatry and Behavioral Sciences, SUNY Downstate Health Sciences University, Brooklyn, New York; 2 VA New York Harbor Healthcare System, Brooklyn

## Abstract

**Importance:**

All of Us is a landmark initiative for population-scale research into a variety of health conditions, including psychiatric disorders.

**Objective:**

To analyze the prevalence, comorbidity, and sociodemographic covariates of psychiatric disorders in the All of Us biobank.

**Design, Setting, and Participants:**

We estimated prevalence, overlap, and sociodemographic correlates for psychiatric disorders as reported in electronic health records for All of Us release 5 (N = 331 380).

**Exposures:**

Social and demographic covariates.

**Main Outcomes and Measures:**

Psychiatric disorders derived from *International Statistical Classification of Diseases, Tenth Revision, Clinical Modification*, codes across 6 broad domains: mood disorders, anxiety disorders, substance use disorders, stress-related disorders, schizophrenia, and personality disorders.

**Results:**

The analytic sample (N = 329 038) was 60.7% female (mean [SD] age, 50.9 [16.8] years). The prevalence of disorders ranged from 11.00% (95% CI, 10.68% to 11.32%) for any mood disorder to less than 1% (eg, obsessive-compulsive disorder, 0.18%; 95% CI, −0.16% to 0.52%), with mood disorders being the most common and personality disorders being the least. There was substantial overlap among disorders, with the majority of participants with a disorder (30 113/58 806, approximately 51%) having 2 or more registered diagnoses and tetrachoric correlations ranging from 0.43 to 0.74. Comparisons of prevalence across demographic categories revealed that non-Hispanic White people, individuals with low socioeconomic status, women and individuals assigned female at birth, and sexual minority individuals are at greatest risk for most disorders.

**Conclusions and Relevance:**

Although rates of disorders among the All of Us cohort are lower than in the general population, considerable variation, comorbidity, and disparities exist across social groups. To improve the practice of equitable precision medicine, researchers can use comprehensive health data from large-scale resources such as All of Us.

## Key Points

**Question:**

What are the prevalence, correlates, and overlap between psychiatric disorders reported in the All of Us Research Program?

**Findings:**

In this cross-sectional study, the prevalence of diagnosed psychiatric disorders ranged from approximately 1% to 11% with personality disorders being the least common and mood disorders being the most common. Associations across sociodemographic factors recapitulated previous epidemiological research.

**Meaning:**

While the prevalence of psychiatric disorders in All of Us was lower than population estimates, the patterns across disorders and sociodemographic characteristics were congruent with prior research.

## Teaser

This cross-sectional study examines electronic health record data from the All of Us Research Program to measure prevalence, correlates, and overlap between psychiatric disorders.

## Introduction

Psychiatric disorders pose a significant burden to public health. Conditions such as depression, anxiety, and substance use disorders are leading contributors to the national burden of disease.^1^ Importantly, these disorders rarely manifest in isolation, showing strong patterns of comorbidity.^2^

To improve diversity in health research, interrogate health disparities, and deliver on the promise of equitable precision medicine, the National Institutes of Health launched the All of Us program, a historic effort to collect and study data from at least 1 million people living in the United States.^3^ Beginning in 2018, participants could complete surveys, provide genotypic data, and link their electronic health records (EHRs) to help build a comprehensive database. The goal of the All of Us program is to better understand how biology, lifestyle, and social determinants come together to affect health.

We characterize the prevalence of psychiatric disorders in the All of Us database, comparing estimates with those from nationally representative samples. In addition, we estimate comorbidity across disorders and sociodemographic disparities to help better understand the participants who make up this sample.

## Methods

### The All of Us Research Program

All of Us is a prospective, nationwide cohort study of the effects of lifestyle, environment, and genomics on health outcomes. Participant recruitment is predominantly done through participating health care organizations and in partnership with Federally Qualified Health Centers. Interested participants can enroll as direct volunteers, visiting community-based enrollment sites. Enrollment, informed consent, and baseline health surveys are done digitally through the All of Us website (https://joinallofus.org).^4^ Participants are then invited to undergo a basic physical examination and biospecimen collection at an affiliated health care site. Participant follow-up is done in 2 ways, passively via linkage with EHRs and actively by periodic follow-up surveys. We included data from participants enrolled between May 6, 2018, and April 1, 2021 (release 5, N = 331 380). Analyses were conducted in accordance with the All of Us Code of Conduct and the institutional review board of SUNY Downstate. Participants provided informed consent when joining All of Us, including authorizing All of Us to access their EHRs.

### Measures

Diagnoses were based on phecodes,^5^ curated groupings of related billing codes from the *International Statistical Classification of Diseases, Tenth Revision, Clinical Modification (ICD-10-CM)* (eTable 1 in the Supplement). We selected all relevant *ICD-10-CM* codes for disorders related to mood, anxiety, substance use, stress, personality, eating, psychotic, and other disorders. Individuals with 2 or more *ICD-10-CM* codes were considered to have a diagnosis, based on prior EHR analyses.^6^ Our analyses also included measures for age, sex, gender identity, sexual orientation, race and ethnicity, educational attainment, household income, access to health insurance, and country of origin (eTable 2 and the eAppendix in the Supplement contain a full description). Race and ethnicity data were provided by All of Us, which used a survey asking how participants identify to categorize responses as Asian; Black or African American; Hispanic or Latino, Latina, or Latinx; non-Hispanic White; multiracial; and other race or ethnicity, for identities that did not fit into any of the other categories.

## Results

Table 1 presents the prevalence for each disorder. Mood disorders were the most common (11.0%; 95% CI, 10.68%-11.32%), followed by anxiety disorders (10.11%; 95% CI, 9.79%-10.44%), substance use disorders (7.22%; 95% CI, 6.89%-7.54%), and stress-related disorders (2.89%; 95% CI, 2.55%-3.23%). Approximately 1% of participants (1.07%; 95% CI, 0.73%-1.41%) had a documented sleep disorder. Attention-deficit/hyperactivity disorder, schizophrenia, personality disorders, eating disorders, and obsessive-compulsive disorder each had a prevalence at or below 1% (Table 1). The prevalence of each disorder was lower than those from nationally representative samples (prevalence by sociodemographic category is provided in eFigure 1 in the Supplement). When we constrained for more rigorous definitions of being affected (≥3 and ≥4 *ICD-10-CM* codes), there was a steady decrease in the prevalence (eAppendix, eTable 3, and eFigure 2 in the Supplement).

**Table 1.** Prevalence of Psychiatric Diagnoses in the All of Us Research Program (N = 329 038)

Phenotype	No. of cases	Prevalence, % (95% CI)	Population prevalence, %^a^
Major depressive disorder	30 544	9.28 (8.96 to 9.61)	16.6
Bipolar	7411	2.25 (1.91 to 2.59)	3.9
Dysthymic disorder	1661	0.50 (0.16 to 0.85)	2.5
Any mood disorder	36 191	11.00 (10.68 to 11.32)	20.8
Anxiety disorder (unspecified)	29 989	9.11 (8.79 to 9.44)	
Generalized anxiety disorder	8320	2.53 (2.19 to 2.87)	5.7
Social anxiety disorder/agoraphobia	2934	0.89 (0.55 to 1.23)	12.1/1.4
Phobia	406	0.12 (−0.22 to 0.46)	12.5
Any anxiety disorder	33 276	10.11 (9.79 to 10.44)	28.8
Alcohol use disorder	6201	1.88 (1.55 to 2.22)	29.1
Tobacco use disorder	17 272	5.25 (4.92 to 5.58)	17.7
Drug use disorder	9907	3.01 (2.67 to 3.35)	9.9
Cannabis	2823	0.86 (0.52 to 1.20)	
Cocaine	3326	1.01 (0.67 to 1.35)	
Opioids	5688	1.73 (1.39 to 2.07)	
Other substances	6826	2.07 (1.74 to 2.41)	
Any substance use disorder	23 742	7.22 (6.89 to 7.54)	32.3
Adjustment disorder	4565	1.39 (1.05 to 1.73)	
Posttraumatic stress disorder	5525	1.68 (1.34 to 2.02)	6.1
Any stress-related disorder	9510	2.89 (2.55 to 3.23)	
Sleep disorder	3511	1.07 (0.73 to 1.41)	5.6
Schizophrenia	2653	0.81 (0.47 to 1.15)	0.9
Attention-deficit/hyperactivity disorder	2475	0.75 (0.41 to 1.09)	8.1
Antisocial personality/borderline personality disorder	894	0.27 (−0.07 to 0.61)	3.8/2.7
Schizotypal personality disorder	45	0.01 (−0.33 to 0.36)	0.6
Any personality disorder	1497	0.45 (0.11 to 0.80)	0.3 to 2.7
Eating disorder, females/males	559	0.17 (−0.17 to 0.51)	8.4/2.2
Obsessive-compulsive disorder	603	0.18 (−0.16 to 0.52)	1.9

^a^ Estimates for population prevalence are described in the eAppendix in the Supplement.

The Figure presents the tetrachoric correlations between diagnoses and patterns of comorbidity. We observed significant correlations across all disorders (*r* = 0.43-0.75). Approximately 51% of the diagnoses (30 113/58 806) involved some overlap. The majority of participants with multiple diagnoses (64%, n = 19 481) had some configuration of mood, anxiety, and substance use disorders. While the frequency of each disorder was lower using more restrictive definitions, the patterns of correlation and comorbidity remained virtually unchanged (eAppendix and eFigures 3 and 4 in the Supplement).

**Figure.**
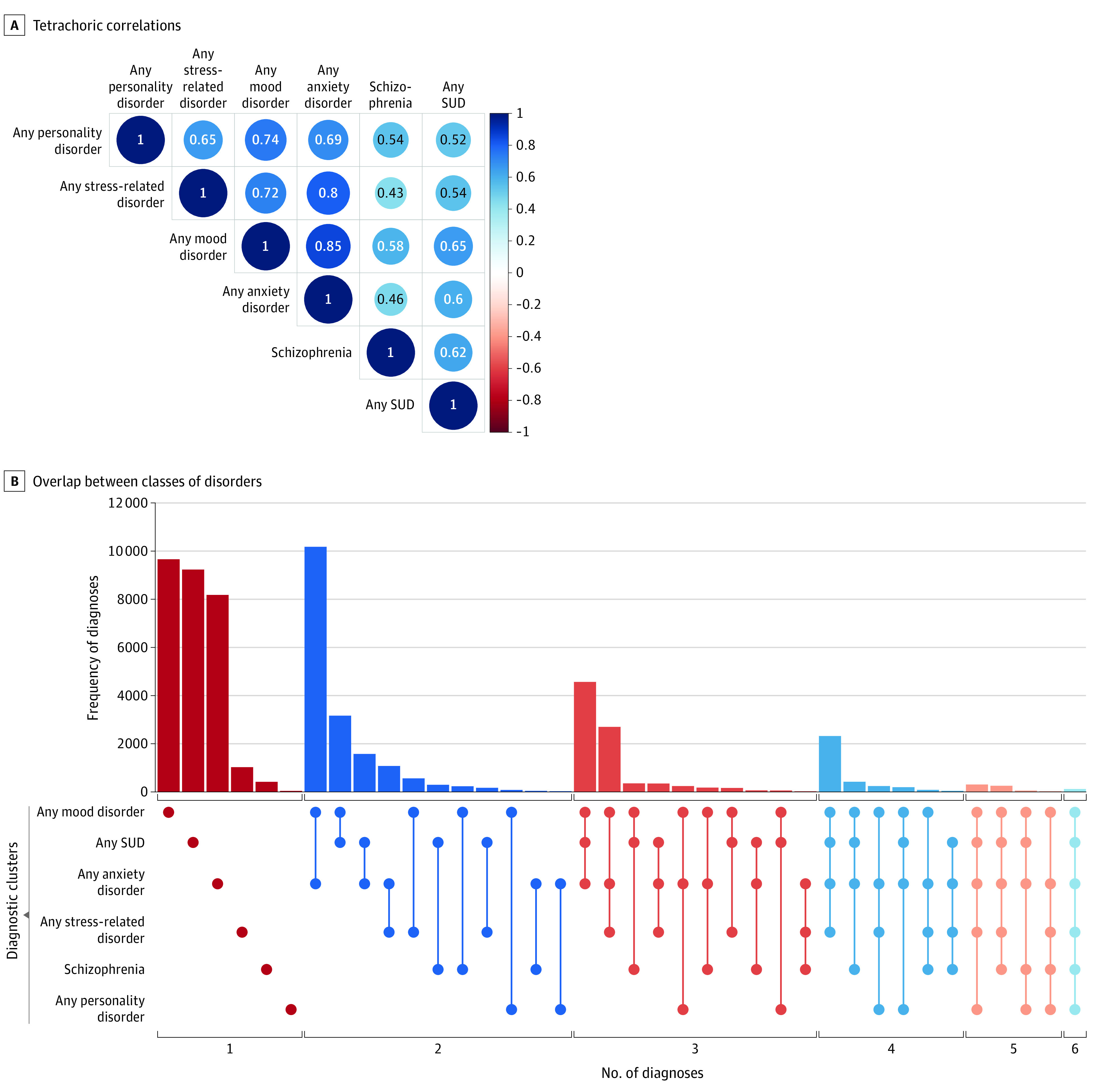
Correlations and Patterns of Comorbidity Across 6 Classes of Disorders All correlations are significant at *P* < .001. SUD indicates substance use disorder.

Table 2 presents the adjusted odds ratios (OR) for sociodemographic factors. Men and those assigned male at birth had lower odds of mood and anxiety disorders (from OR, 0.78 [95% CI, 0.68-0.90] to OR, 0.81 [95% CI, 0.71-0.93]), but increased risk for substance use disorders and schizophrenia (from OR, 1.27 [95% CI, 1.09-1.47] to OR, 1.82 [95% CI, 1.25-2.65]). Participants who were lesbian, gay, bisexual, transgender, or queer (LGBTQ) were at increased risk for most disorders (from OR, 1.18 [95% CI, 1.13-1.23] to OR, 1.88 [95% CI, 1.66-2.13]). People from all racial and ethnic categories were at reduced risk for most disorders compared with non-Hispanic White participants (from OR, 0.30 [95% CI, 0.26-0.35] to OR, 0.86 [95% CI, 0.78-0.93]), except for schizophrenia, where participants who were Black or African American, multiracial, or another race and ethnicity were at increased risk (from OR, 1.22 [95% CI, 1.10-1.36] to OR, 1.72 [95% CI, 1.30-2.28]). Participants born outside the United States were at reduced risk for every disorder (OR, 0.36 [95% CI, 0.34-0.38] to OR, 0.67 [95% CI, 0.64-0.70]). Participants from each educational and income category were at lower risk relative to those with less than a high school education or those reporting an annual income less than $25 000, respectively (from OR, 0.04 [95% CI, 0.02-0.07] to OR, 0.88 [95% CI, 0.81-0.96]). Participants from each relationship category were at increased risk relative to those who were married (from OR, 1.10 [95% CI, 1.04-1.15] to OR, 2.47 [95% CI, 2.00-3.04]). Results were similar when using more restrictive definitions of diagnosis (eAppendix and eTables 4 and 5 in the Supplement).

**Table 2.** Adjusted Estimates of Risk for Psychiatric Disorders

	Any mood disorder		Any anxiety disorder		Any substance use disorder		Any stress-related disorder		Schizophrenia		Any personality disorder	
	aOR (SE)^a^	95% CI	aOR (SE)^a^	95% CI	aOR (SE)^a^	95% CI	aOR (SE)^a^	95% CI	aOR (SE)^a^	95% CI	aOR (SE)^a^	95% CI
Sex												
Female	1 [Reference]		1 [Reference]		1 [Reference]		1 [Reference]		1 [Reference]		1 [Reference]	
Male	0.81 (0.07)^b^	0.71-0.93	0.84 (0.07)	0.73-0.96	1.30 (0.08)^b^	1.12-1.51	0.79 (0.12)	0.63-0.99	1.04 (0.19)	0.72-1.50	0.85 (0.23)	0.54-1.34
Neither selected	0.98 (0.06)	0.87-1.09	0.98 (0.06)	0.87-1.11	1.22 (0.07)^b^	1.07-1.39	0.90 (0.10)	0.74-1.09	1.09 (0.18)	0.77-1.54	0.63 (0.28)	0.36-1.10
Gender												
Woman	1 [Reference]		1 [Reference]		1 [Reference]		1 [Reference]		1 [Reference]		1 [Reference]	
Man	0.86 (0.07)	0.76-0.98	0.78 (0.07)^b^	0.68-0.90	1.27 (0.08)^b^	1.09-1.47	1.14 (0.12)	0.91-1.44	1.82 (0.19)^b^	1.25-2.65	0.90 (0.24)	0.56-1.43
Neither selected	1.00 (0.05)	0.92-1.10	0.94 (0.05)	0.86-1.04	1.09 (0.06)	0.97-1.22	1.32 (0.08)^b^	1.13-1.53	1.58 (0.15)^b^	1.18-2.11	1.48 (0.16)	1.08-2.02
Sexual orientation												
Heterosexual	1 [Reference]		1 [Reference]		1 [Reference]		1 [Reference]		1 [Reference]		1 [Reference]	
Other than heterosexual	1.33 (0.02^b^	1.29-1.38	1.23 (0.02)^b^	1.19-1.27	1.18 (0.02)^b^	1.13-1.23	1.37 (0.03)^b^	1.30-1.46	1.56 (0.05)^b^	1.41-1.72	1.88 (0.06)^b^	1.66-2.13
Age, y												
18-29	1 [Reference]		1 [Reference]		1 [Reference]		1 [Reference]		1 [Reference]		1 [Reference]	
30-44	1.67 (0.02)^b^	1.60-1.74	1.53 (0.02)^b^	1.46-1.59	2.23 (0.03)^b^	2.12-2.35	1.61 (0.04)^b^	1.50-1.74	2.39 (0.08)^b^	2.06-2.77	1.26 (0.08)^b^	1.08-1.47
45-64	1.99 (0.02)^b^	1.91-2.08	1.51 (0.02)^b^	1.45-1.57	2.39 (0.03)^b^	2.27-2.52	1.69 (0.04)^b^	1.56-1.82	2.44 (0.08)^b^	2.10-2.83	0.99 (0.08)	0.85-1.17
≥65	1.55 (0.02)^b^	1.48-1.63	1.09 (0.02)^b^	1.04-1.14	1.23 (0.03)^b^	1.16-1.31	1.18 (0.04)^b^	1.08-1.29	1.03 (0.10)	0.84-1.26	0.35 (0.12)^b^	0.28-0.44
Race and ethnicity												
Asian	0.46 (0.05)^b^	0.42-0.51	0.46 (0.05)^b^	0.42-0.51	0.42 (0.09)^b^	0.35-0.50	0.62 (0.09)^b^	0.52-0.74	0.56 (0.29)	0.32-0.98	0.28 (0.32)^b^	0.15-0.52
Black and African American	0.51 (0.02)^b^	0.50-0.53	0.41 (0.02)^b^	0.39-0.42	0.67 (0.02)^b^	0.65-0.69	0.66 (0.03)^b^	0.62-0.70	1.22 (0.05)^b^	1.10-1.36	0.30 (0.08)^b^	0.26-0.35
Hispanic or Latino	0.74 (0.02)^b^	0.71-0.77	0.69 (0.02)^b^	0.66-0.71	0.77 (0.02)^b^	0.73-0.81	0.76 (0.04)^b^	0.71-0.82	1.13 (0.07)	0.98-1.30	0.47 (0.09)^b^	0.39-0.55
Non-Hispanic White	1 [Reference]		1 [Reference]		1 [Reference]		1 [Reference]		1 [Reference]		1 [Reference]	
Multiracial	0.89 (0.05)	0.82-0.98	0.82 (0.05)^b^	0.75-0.90	1.01 (0.06)	0.90-1.12	1.04 (0.08)	0.89-1.21	1.72 (0.14)^b^	1.30-2.28	0.92 (0.17)	0.66-1.27
Not reported	0.85 (0.04)^b^	0.78-0.92	0.80 (0.04)^b^	0.74-0.87	1.00 (0.05)	0.91-1.09	0.89 (0.08)	0.77-1.04	1.55 (0.12)^b^	1.23-1.95	0.67 (0.18)	0.47-0.95
Other race and ethnicity	0.92 (0.04)	0.84-1.00	0.86 (0.04)^b^	0.78-0.93	1.09 (0.05)	0.99-1.21	1.07 (0.08)	0.92-1.24	1.52 (0.14)^b^	1.16-1.99	0.83 (0.18)	0.59-1.18
Country of origin												
US born	1 [Reference]		1 [Reference]		1 [Reference]		1 [Reference]		1 [Reference]		1 [Reference]	
Not US born	0.67 (0.02)^b^	0.64-0.70	0.61 (0.02)^b^	0.58-0.64	0.36 (0.03)^b^	0.34-0.38	0.62 (0.04)^b^	0.57-0.67	0.46 (0.09)^b^	0.38-0.55	0.50 (0.12)^b^	0.40-0.64
Not reported	0.94 (0.06)	0.84-1.05	0.91 (0.06)	0.81-1.02	1.09 (0.06)	0.97-1.22	0.90 (0.10)	0.73-1.10	1.12 (0.14)	0.84-1.48	0.93 (0.25)	0.57-1.51
Education												
Less than HS	1 [Reference]		1 [Reference]		1 [Reference]		1 [Reference]		1 [Reference]		1 [Reference]	
HS diploma or equivalent	0.95 (0.02)	0.91-0.99	0.97 (0.02)	0.93-1.02	0.84 (0.02)^b^	0.80-0.87	1.05 (0.04)	0.97-1.13	0.79 (0.05)^b^	0.71-0.88	0.86 (0.09)	0.72-1.03
Some college	0.95 (0.02)	0.91-0.99	0.98 (0.02)	0.94-1.03	0.66 (0.02)^b^	0.63-0.69	1.06 (0.04)	0.99-1.15	0.58 (0.06)^b^	0.51-0.65	0.97 (0.09)	0.81-1.16
College degree or higher	0.67 (0.02)^b^	0.64-0.70	0.71 (0.02)^b^	0.68-0.75	0.26 (0.03)^b^	0.25-0.28	0.78 (0.04)^b^	0.72-0.85	0.36 (0.08)^b^	0.30-0.42	0.52 (0.11)^b^	0.42-0.64
Not reported	0.88 (0.04)^b^	0.81-0.96	0.84 (0.05)^b^	0.76-0.92	0.90 (0.04)	0.83-0.98	0.85 (0.08)	0.73-0.99	0.81 (0.10)	0.66-0.99	0.90 (0.19)	0.62-1.29
Income, $/y												
<25 000	1 [Reference]		1 [Reference]		1 [Reference]		1 [Reference]		1 [Reference]		1 [Reference]	
25 000-50 000	0.67 (0.02)^b^	0.65-0.69	0.74 (0.02)^b^	0.71-0.77	0.56 (0.02)^b^	0.54-0.59	0.68 (0.03)^b^	0.63-0.72	0.36 (0.08)^b^	0.31-0.43	0.49 (0.08)^b^	0.41-0.57
50 000-75 000	0.54 (0.02)^b^	0.52-0.56	0.61 (0.02)^b^	0.59-0.64	0.36 (0.03)^b^	0.33-0.38	0.59 (0.04)^b^	0.55-0.64	0.16 (0.15)^b^	0.12-0.22	0.33 (0.12)^b^	0.26-0.42
75 000-100 000	0.45 (0.03)^b^	0.43-0.47	0.54 (0.03)^b^	0.51-0.57	0.29 (0.04)^b^	0.26-0.31	0.46 (0.05)^b^	0.41-0.50	0.10 (0.24)^b^	0.06-0.15	0.26 (0.16)^b^	0.19-0.35
>100 000	0.33 (0.02)^b^	0.31-0.34	0.40 (0.02)^b^	0.39-0.42	0.18 (0.04)^b^	0.17-0.20	0.36 (0.04)^b^	0.33-0.40	0.04 (0.26)^b^	0.02-0.07	0.12 (0.16)^b^	0.09-0.16
Not reported	0.68 (0.02)^b^	0.66-0.70	0.73 (0.02)^b^	0.71-0.76	0.72 (0.02)^b^	0.69-0.75	0.71 (0.03)^b^	0.67-0.76	0.74 (0.05)^b^	0.68-0.82	0.66 (0.07)^b^	0.57-0.76
Health insurance												
Has	1 [Reference]		1 [Reference]		1 [Reference]		1 [Reference]		1 [Reference]		1 [Reference]	
Does not have	0.41 (0.03)^b^	0.39-0.44	0.40 (0.03)^b^	0.38-0.43	0.41 (0.03)^b^	0.38-0.43	0.53 (0.05)^b^	0.48-0.58	0.28 (0.09)^b^	0.24-0.34	0.55 (0.11)^b^	0.44-0.68
Not reported	0.69 (0.04)^b^	0.64-0.74	0.67 (0.04)^b^	0.62-0.73	0.76 (0.04)^b^	0.71-0.82	0.72 (0.07)^b^	0.63-0.83	0.69 (0.10)^b^	0.56-0.83	0.57 (0.18)^b^	0.40-0.81
Marital information												
Married	1 [Reference]		1 [Reference]		1 [Reference]		1 [Reference]		1 [Reference]		1 [Reference]	
Cohabiting	1.13 (0.03)^b^	1.08-1.19	1.10 (0.03)^b^	1.04-1.15	1.85 (0.03)^b^	1.74-1.97	1.14 (0.05)^b^	1.04-1.25	1.59 (0.10)^b^	1.30-1.95	1.24 (0.12)	0.99-1.56
Divorced	1.41 (0.02)^b^	1.36-1.46	1.29 (0.02)^b^	1.25-1.34	1.88 (0.02)^b^	1.80-1.97	1.48 (0.03)^b^	1.39-1.58	2.08 (0.08)^b^	1.79-2.43	2.00 (0.09)^b^	1.69-2.37
Never married	1.24 (0.02)^b^	1.20-1.29	1.10 (0.02)^b^	1.06-1.14	1.70 (0.02)^b^	1.63-1.78	1.17 (0.03)^b^	1.10-1.25	2.46 (0.07)^b^	2.12-2.84	1.55 (0.09)^b^	1.31-1.83
Separated	1.40 (0.03)^b^	1.32-1.48	1.24 (0.03)^b^	1.16-1.32	1.93 (0.03)^b^	1.81-2.06	1.40 (0.05)^b^	1.26-1.55	2.09 (0.10)^b^	1.72-2.54	1.87 (0.13)^b^	1.45-2.41
Widowed	1.32 (0.03)^b^	1.26-1.39	1.17 (0.03)^b^	1.11-1.24	1.73 (0.03)^b^	1.61-1.85	1.47 (0.05)^b^	1.34-1.61	1.90 (0.11)^b^	1.52-2.37	1.38 (0.15)	1.03-1.86
Not reported	1.22 (0.04)^b^	1.13-1.31	1.15 (0.04)^b^	1.06-1.24	1.76 (0.04)^b^	1.62-1.90	1.36 (0.06)^b^	1.20-1.55	2.47 (0.11)^b^	2.00-3.04	2.00 (0.15)^b^	1.50-2.66

Abbreviations: aOR, adjusted odds ratio; HS, high school.

^a^ All estimates conditional on all other covariates included in the model.

^b^ *P* < .05 ÷ 6 = .008.

## Discussion

We explored psychiatric disorders in the All of Us research program, examining prevalence, comorbidity, and sociodemographic disparities and how they vary across *ICD-10-CM* thresholds for defining who is affected. Other biobanks have a well-known “healthy participant” bias.^7^ This bias is apparent in All of Us given that disorders captured in the EHR occur at lower frequency than estimates from nationally representative samples.^2,8^ In addition, the positive association between health insurance and each disorder suggests some barrier to health care for participants without insurance, similar to prior research.^9^ Diagnoses were strongly intercorrelated, supporting the idea that psychiatric disorders may in part share common causes.^10^ Most respondents with any registered diagnosis had diagnoses for 2 or more disorders in their EHR. The most common configuration of overlap involved mood, anxiety, and substance use disorders. These patterns of comorbidity remained consistent, even for more restrictive definitions.

Disparities across sociodemographic factors were similar to disparities from nationally representative samples.^2^ Participants who were assigned female at birth, women, and LGBTQ individuals were at increased risk of most disorders.^8^ People from every racial and ethnic category were at reduced risk of most disorders, relative to non-Hispanic White participants, a commonly observed pattern that is in the opposite direction of physical health disparities.^11^ One exception was the increased risk of schizophrenia for Black and African American, multiracial, and other non-White participants, which has previously been attributed as a consequence of racism^12^ and bias in the diagnosis.^13^ Further, participants with a college degree and annual household incomes more than $100 000 were at the lowest risk for each disorder, which could reflect processes of social causation^14^ or social selection or drift.^15^ Again, these patterns remained consistent regardless of the number of *ICD-10-CM* codes required for diagnosis.

### Limitations

Our analysis has several limitations. We focused on participants with any documented history of disorders. Our measures of diagnosis could be biased in that (1) they are limited to those who have sought treatment and (2) they could reflect misdiagnosis given that they require only 2 records in the EHR. Sensitivity analyses using more stringent thresholds resulted in a lower estimated prevalence for each disorder. Future work can leverage available genetic data in All of Us to examine whether there are distinct causes across different thresholds for inclusion.

## Conclusions

The goal of the All of Us program is to create a resource for medical research in the United States, with an emphasis in improving health equity and representation. In the current analysis, we investigated the available data on psychiatric disorders. Although the rates of disorders in the All of Us cohort were lower than in the general population, considerable variation, comorbidity, and disparities existed across social groups. Future research can draw on the vast genetic, lifestyle, and other social data to investigate the causes and consequences of psychiatric conditions.

## Notes

Supplement.
eAppendix
**eTable 1.** ICD-10-CM codes and corresponding phecodes **eTable 2.** All of Us sample characteristics (N = 329,038) **eTable 3.** Prevalence across ICD-10-CM code thresholds **eTable 4.** Adjusted estimates of risk for psychiatric disorders (3+ ICD codes) **eTable 5.** Adjusted estimates of risk for psychiatric disorders (4+ ICD codes) **eFigure 1.** Prevalence of psychiatric disorders across sociodemographic risk factors **eFigure 2.** Prevalence of disorders across ICD thresholds **eFigure 3.** Comorbidity of disorders across ICD thresholds **eFigure 4.** Tetrachoric correlations across those with (A) 3+ and (B) 4+ ICD codes
