# Supplementary material for: Prevalence, Comorbidity, and Sociodemographic Correlates of Psychiatric Disorders Reported in the All of Us Research Program
Source: JAMA Psychiatry. 2022 Apr 20;79(6):622–8. doi: 10.1001/jamapsychiatry.2022.0685 (PMC9021986; doi:10.1001/jamapsychiatry.2022.0685)
Supplement: Supplement. — eAppendix eTable 1. ICD-10-CM codes and corresponding phecodes eTable 2. All of Us sample characteristics (N = 329,038) eTable 3. Prevalence across ICD-10-CM code thresholds eTable 4. Adjusted estimates of risk for psychiatric disorders (3+ ICD codes) eTable 5. Adjusted estimates of risk for psychiatric disorders (4+ ICD codes) eFigure 1. Prevalence of psychiatric disorders across sociodemographic risk factors eFigure 2. Prevalence of disorders across ICD thresholds eFigure 3. Comorbidity of disorders across ICD thresholds eFigure 4. Tetrachoric correlations across those with (A) 3+ and (B) 4+ ICD codes [file jamapsychiatry-e220685-s001.pdf]

Supplemental Online Content

Barr PB, Bigdeli TB, Meyers JL. Prevalence, comorbidity, and sociodemographic correlates of psychiatric disorders reported in the All of Us research program. *JAMA Psychiatry*. Published online April 20, 2022. doi:10.1001/jamapsychiatry.2022.0685

eAppendix

1 STUDY INTRODUCTION **2**

2 THE ALL OF US BIOBANK **3**

2.1 PSYCHIATRIC DIAGNOSES 3
2.2 SOCIODEMOGRAPHIC RISK FACTORS 4

3 SENSITIVITY ANALYSES OF EHR-BASED PHENOTYPES **6**

4 SOURCES FOR PREVALENCE OF PSYCHIATRIC DISORDERS **10**

eREFERENCES **11**

ETABLE 1: ICD10CM CODES AND CORRESPONDING PHECODES **14**

ETABLE 2: ALL OF US SAMPLE CHARACTERISTICS (N = 329,038) **35**

ETABLE 3: PREVALENCE ACROSS ICD10CM CODE THRESHOLDS **36**

ETABLE 4: ADJUSTED ESTIMATES OF RISK FOR PSYCHIATRIC DISORDERS (3+ ICD CODES) **37**

ETABLE 5: ADJUSTED ESTIMATES OF RISK FOR PSYCHIATRIC DISORDERS (4+ ICD CODES) **39**

eFIGURE 1: PREVALENCE OF PSYCHIATRIC DISORDERS ACROSS SOCIODEMOGRAPHIC RISK FACTORS 5
eFIGURE 2: PREVALENCE OF DISORDERS ACROSS ICD THRESHOLDS 7
eFIGURE 3: COMORBIDITY OF DISORDERS ACROSS ICD THRESHOLDS 8
eFIGURE 4: TETRACHORIC CORRELATIONS ACROSS THOSE WITH: (A) 3+ AND (B) 4+ ICD CODES 9

This supplemental material has been provided by the authors to give readers additional information about their work.

© 2022 Barr PB et al. *JAMA Psychiatry*

eAppendix

1 STUDY INTRODUCTION

Psychiatric and substance use disorders remain an ever-present challenge to public health, incurring significant cost to affected individuals, their families, and society. Mental illness is estimated to contribute ~13% to the global burden of disease[1]. Conditions such as depression, anxiety, and substance use disorders are leading contributors to the burden of disease in the United States[2]. Psychiatric and substance use disorders also incur significant financial cost. In the United states, the estimated annual cost ranges from ~$320 billion for serious mental illnesses[3] to ~$78 billion for opioid use[4]. Importantly, these disorders rarely manifest in isolation, showing strong patterns of comorbidity and shared risk factors[5–7].

Historically, estimating the prevalence of psychiatric disorders has relied on nationally representative or population-based surveys[8–10], or through clinical ascertainment[11]. More recently, large-scale biobanks and healthcare systems are becoming increasingly used for research purposes. To date, resources such as the UK Biobank[12], the Million Veteran Program[13], Biobank Japan[14], and FinnGen, among others, have proved to be an invaluable resource in the understanding of the causes of various psychiatric and related disorders. While valuable, these resources may not reflect the unique context within the US. Additionally, much of the research conducted in this area has focused primarily on individuals of European ancestries[15,16]. This is despite the previously observed differences in rates and patterns of psychiatric disorders by race-ethnicity, as well as known risk factors that are especially pertinent in the US given its sordid history with racism and inequality. In order to improve diversity in health-focused epidemiological research, further interrogate existing health disparities, and deliver on the promise of equitable precision medicine, the National Institutes of Health launched the All of Us program, a historic effort to collect and study data from at least one million people living in the United States[17]. Beginning in 2018, participants were able to complete surveys, provide genotypic data, and link their electronic health records to help build a comprehensive database of their health-related data. The goal of the All of Us program is to use this data to understand how biology, lifestyle and social determinants come together to affect health, and ultimately to treat and prevent illness.

The current analysis focuses on characterizing the prevalence, comorbidity, and sociodemographic disparities for psychiatric and substance use disorders in the All of Us database. We compare these estimates to those from nationally representative and population-based samples to help better understand the etiology of psychiatric disorders in All of Us, and the participants that make up this new sample.

2 THE ALL OF US BIOBANK

The All of Us Research Program is a prospective cohort study aiming to recruit at least one million individuals in the United States, with the overall goal of providing a unique resource to study the effects of lifestyle, environment and genomics on health and health outcomes. Participant recruitment is predominantly done through participating health care provider organizations and in partnership with Federally Qualified Health Centers, with an emphasis on recruiting persons affiliated with those centers. Interested potential participants can also enroll in the program as direct volunteers, visiting community-based enrollment sites. Initial enrollment, informed consent (including consent to share EHRs), and baseline health surveys are done digitally through the All of Us program website (https://joinallofus.org). Once this step is completed, the participant is invited to undergo a basic physical exam and biospecimen collection at the affiliated healthcare site. Participant follow-up is done in two ways, passively via linkage with EHR and actively by periodic follow-up surveys. For this study, we included data from participants enrolled in the study between May 6, 2018 and April 1st, 2021 (All of Us release 5, N = 331,380). This work was performed on data collected by the previously described All of Us Research Program using the All of Us Researcher Workbench, a cloud-based platform where approved researchers can access and analyze All of Us data. All analyses were in concordance with the ethical guidelines outlined in the All of Us Code of Conduct and the IRB at SUNY Downstate.

2.1 Psychiatric Diagnoses

We derived diagnoses based on phecodes from registered ICD10CM codes in the All of Us system. Individuals with 2+ ICD codes were considered as having a diagnosis, based on prior analyses in electronic health systems[18]. First, we selected all relevant ICD codes for the more prevalent psychiatric disorders. We then clustered these ICD codes using phecodes[19] from the *PheWAS* package in R[20]. Phecodes provide a convenient way to cluster codes in EHR based data[21] and have been validated previously[21,22]. A full list of the EHR descriptions, ICD10CM codes, and corresponding phecodes is provided in eTable 1. We focused our main analyses on the following broad clusters of psychiatric disorders:

- Any mood disorder (MOOD): those with a registered diagnosis of major depressive disorder, dysthymic disorder, or bipolar disorder.
- Any anxiety disorder (ANX) : those with a registered diagnosis generalized anxiety disorder, social anxiety/agoraphobia, any phobia, or anxiety disorder (unspecified).

- Any substance use disorder (SUD): those with a registered diagnosis of an alcohol use disorder or other substance use disorder.
- Any stress-related disorder (STRESS): those with a registered diagnosis of post-traumatic stress disorder or an adjustment disorder.
- Schizophrenia (SCZ): those with any registered diagnosis of schizophrenia.
- Any personality disorder (PERS): those with a registered diagnosis of antisocial, borderline, or schizotypal personality disorder.

2.2 Sociodemographic Risk Factors

All of Us provides extensive measures of social and demographic characteristics. Where possible, we tried to match characteristics to other nationally representative samples[8]. A full breakdown of the sample characteristics is presented in eTable 2. Our analyses included the following sociodemographic risk factors (as defined by the All of Us database):

- Age: ages 18-29 (reference), ages 30-44, ages 45-64, ages 65 and older
- Sex assigned at birth: female (reference), male, and those who selected neither male nor female
- Gender identity: woman (reference) man, and those who selected neither man nor women
- Sexual orientation: those who identify as straight/heterosexual (reference) vs. all others
- Race-ethnicity: non-Hispanic White (reference), Black/African American, Hispanic or Latino/a/x, Asian, other race-ethnicity, and those who identify as multiracial
- Educational attainment: less than high school (reference), high school diploma or equivalent, some college, and college degree or more
- Annual household income: under $25K annually (reference), $25K-$50K annually, $50K-$75K annually, $75K-$100K annually, and $100K or more annually
- Access to health insurance: those who report having insurance (reference) vs those who report no insurance
- Country of origin: US born (reference) vs foreign born

The raw prevalence for each group of disorder (mood, anxiety, substance, stress-related, schizophrenia, and personality) across each of the sociodemographic covariates is presented in eFigure 1.

© 2022 Barr PB et al. *JAMA Psychiatry*

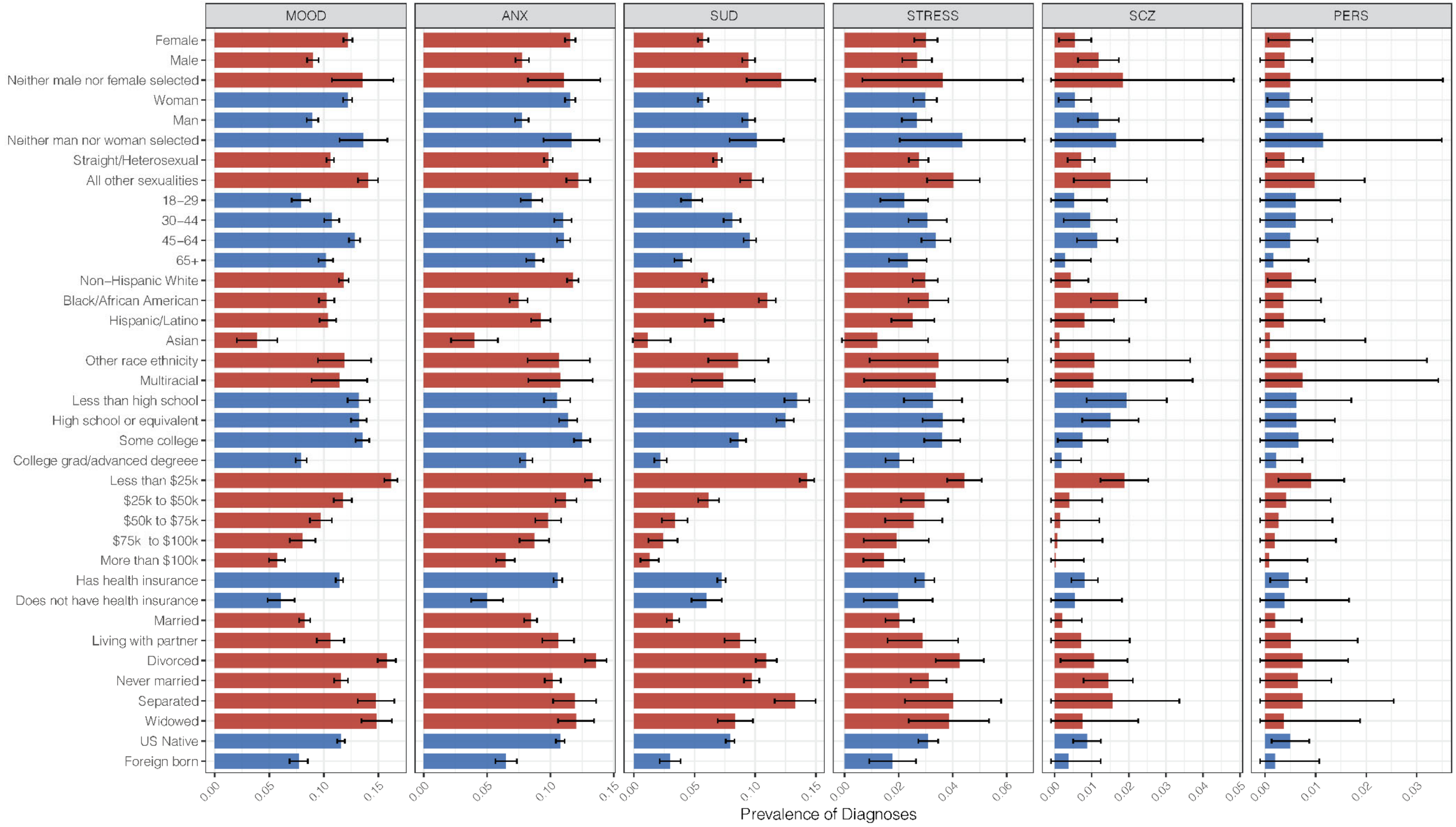

eFigure 1: Prevalence of Psychiatric Disorders across Sociodemographic Risk Factors

© 2022 Barr PB et al. *JAMA Psychiatry*

3 SENSITIVITY ANALYSES OF EHR-BASED PHENOTYPES

Using electronic health records (EHR) for research is a rapidly advancing area. Prior work in medical informatics has shown that using EHR based diagnoses and other administrative data to derive cases can vary across the type of disorder one considers, with more severe disorders generally demonstrating the most reliable measures[23]. Previous work in the PsycheMERGE consortium has used the standard of two or more registered ICD codes as the definition for being considered a “case”[18].

In order to determine whether more stringent definitions of being a “case” altered the prevalence of psychiatric problems in the All of Us database, we ran a series of sensitivity analyses. We restricted the definition of having disorder from 1 or more registered ICD codes and up to 4 or more registered ICD codes. Across all disorders, the total number of “cases” (not mutually exclusive) dropped as thresholds became more restrictive (Total cases 1+ ICD code(s) = 202,422; Total cases 2+ ICD codes = 137,265, Total cases 3+ ICD codes = 106,737; Total cases 4+ ICD codes 88,093). eFigure 2 presents the drop in those meeting criteria of being a “case” as the threshold becomes more restrictive for each disorder included in the analysis. Overall, each disorder follows a similar pattern regardless of the prevalence of that disorder (see eTable 3 for exact counts by disorder). Additionally, when we look at the patterns of comorbidity across ICD thresholds (eFigure 3), we see that these patterns are largely unchanged from using any diagnosis, with the exception that mood disorders became slightly more prevalent than SUD for those with only one registered diagnosis. The main difference is related to the difference in the raw prevalence across thresholds above: as we are more restrictive, fewer participants meet criteria for a given disorder. Regardless of the threshold for defining cases, we see that the correlation between disorders is approximately the same (eFigure 4). Finally, estimates for risk across sociodemographic covariates is relatively stable to inclusion threshold (see eTable4 and eTable5).

Use of diagnoses based on single ICD codes represent a “best-case scenario” for those interested in psychiatric disorders in the All of Us biobank. We have no “gold standard” measurement (such as a physician chart review or Structured Clinical Interview for DSM Disorders confirmed diagnoses[24]) to validate each threshold. However, future research can leverage the genetic data to compare genetic correlations across thresholds with results from published genome wide association studies (GWAS) of confirmed cases. For example, research with the Alcohol Use Disorder Identification Test (AUDIT), has followed this approach and identified AUDIT thresholds that are most likely to capture those who meet criteria for AUD[25].

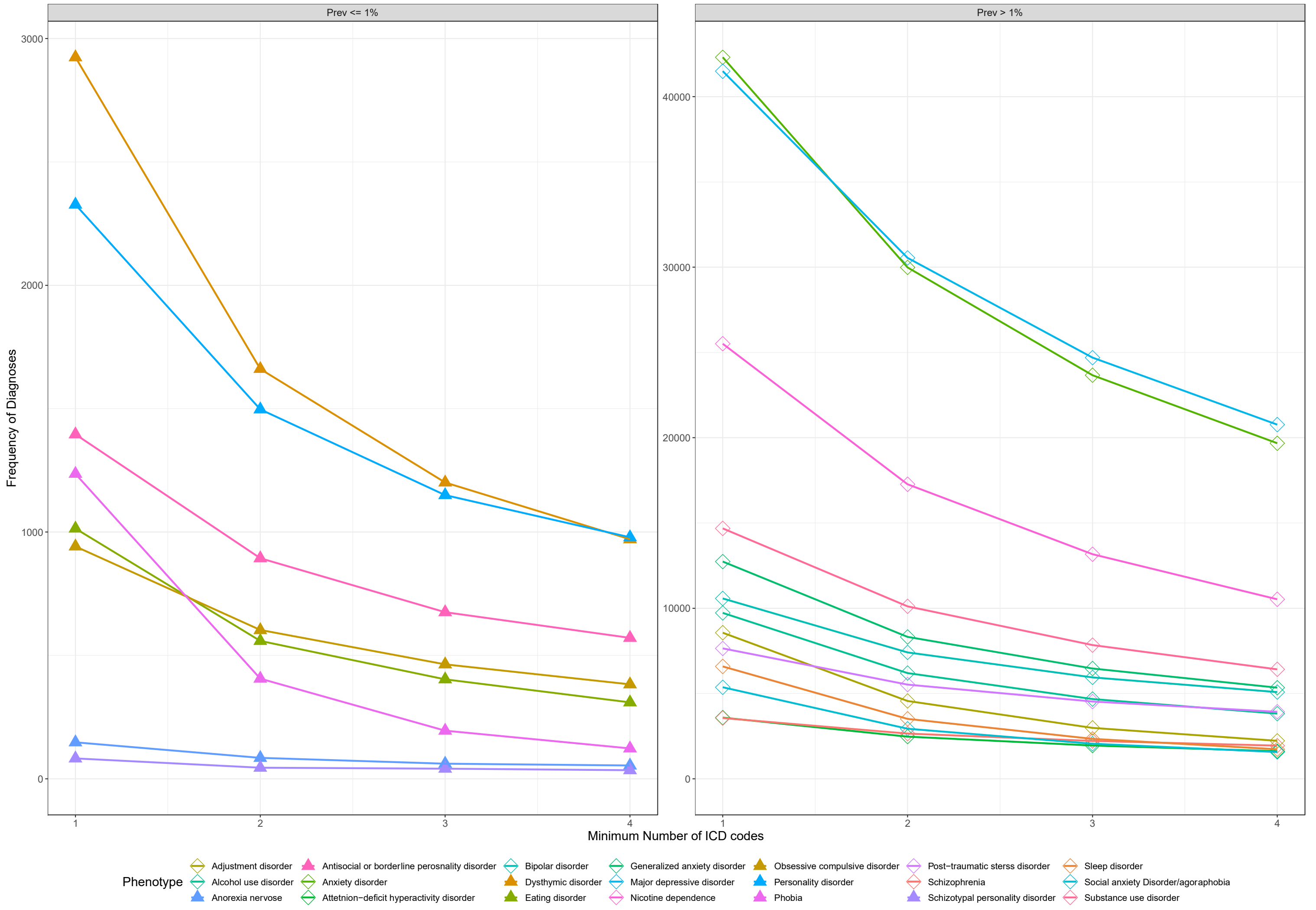

eFigure 2: Prevalence of Disorders Across ICD Thresholds

© 2022 Barr PB et al. *JAMA Psychiatry*

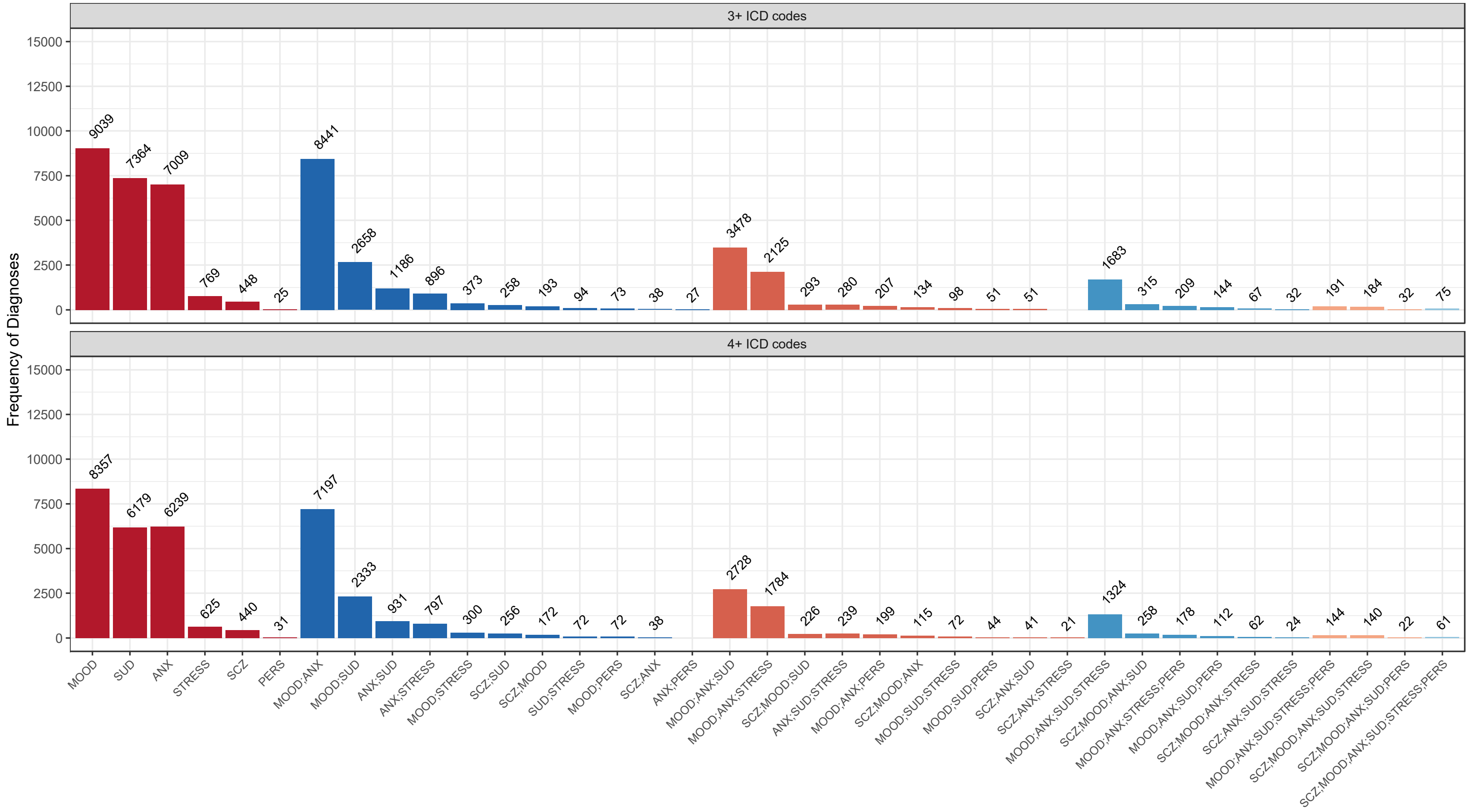

eFigure 3: Comorbidity of Disorders Across ICD Thresholds

© 2022 Barr PB et al. *JAMA Psychiatry*

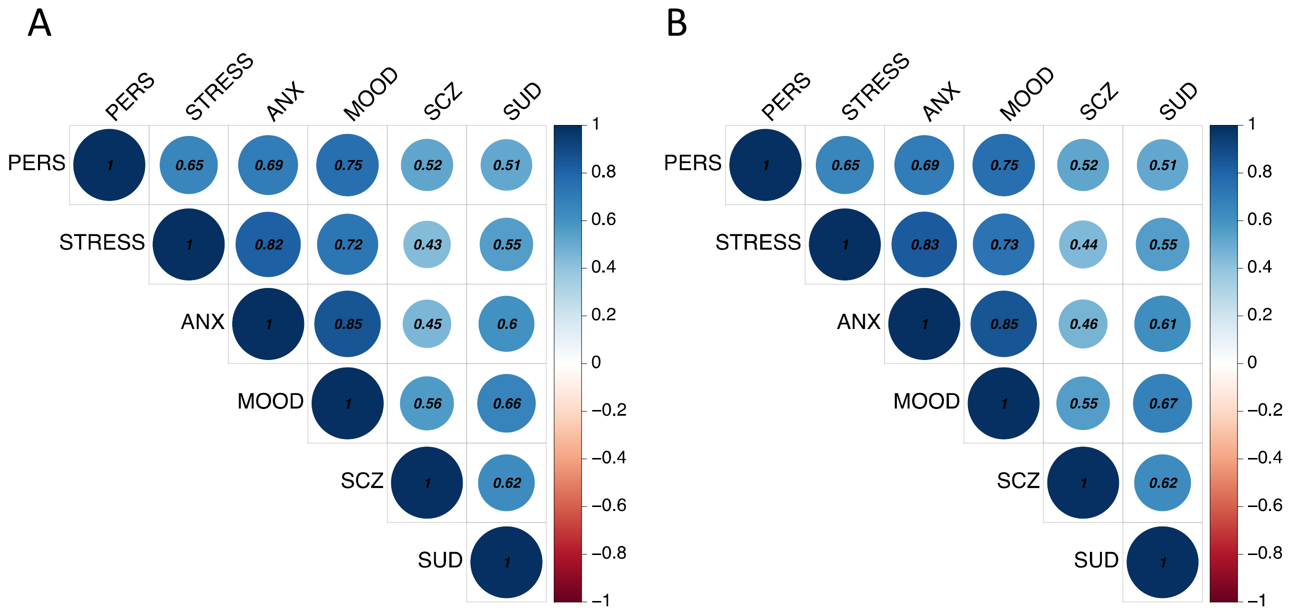

eFigure 4: Tetrachoric Correlations Across Those with: (A) 3+ and (B) 4+ ICD Codes

© 2022 Barr PB et al. *JAMA Psychiatry*

4 SOURCES FOR PREVALENCE OF PSYCHIATRIC DISORDERS

The prevalence of each disorder is lower than the those from nationally representative samples[6–8,26–31]. Below are the sources for estimates of prevalence in the general population.

Phenotype	Population Prevalence	DOI	Reference
Major depressive disorder	16.6%	10.1001/archpsyc.62.6.593	31
Bipolar	3.9%	10.1001/archpsyc.62.6.593	31
Dysthymic disorder	2.5%	10.1001/archpsyc.62.6.593	31
Any mood disorder	20.8%	10.1001/archpsyc.62.6.593	31
Generalized anxiety disorder	5.7%	10.1001/archpsyc.62.6.593	31
Social anxiety disorder/Agoraphobia	12.1/1.4%	10.1001/archpsyc.62.6.593	31
Phobia	12.5%	10.1001/archpsyc.62.6.593	31
Any anxiety disorder	28.8%	10.1001/archpsyc.62.6.593	31
Alcohol use disorder	29.1%	10.1001/jamapsychiatry.2015.0584	6
Drug use disorder	9.9%	10.1001/jamapsychiatry.2015.2132	7
Tobacco use disorder	17.7%	10.1007/s00127-015-1088-0	8
Any substance use disorder	32.3%	10.1007/s00127-015-1088-0	8
Post-traumatic stress disorder	6.1%	10.1007/s00127-016-1208-5	30
Sleep disorder	5.6%	10.1016/S0022-3956(97)00002-2	28
Schizophrenia	0.9%	10.1001/archpsyc.64.1.19	29
Attention-deficit/hyperactivity disorder	8.1%	10.1001/archpsyc.62.6.593	31
Antisocial personality/Borderline personality disorder	3.8/2.7%	10.1521/pedi.2010.24.4.412	27
Shizotypal personality disorder	0.6%	10.1521/pedi.2010.24.4.412	27
Any personality disorder	0.3 - 2.7%	10.1521/pedi.2010.24.4.412	27
Eating Disorder	8.4/2.2%	10.1093/ajcn/nqy342	26
Obsessive compulsive disorder	1.9%	10.1521/pedi.2010.24.4.41	27

© 2022 Barr PB et al. *JAMA Psychiatry*

5 EREFERENCES

1. Vigo D, Thornicroft G, Atun R. Estimating the true global burden of mental illness. *The Lancet Psychiatry*. 2016;3(2):171-178. doi:10.1016/S2215-0366(15)00505-2
2. Murray CJL, Mokdad AH, Ballestros K, et al. The state of US health, 1990-2016: Burden of diseases, injuries, and risk factors among US states. *JAMA - J Am Med Assoc*. 2018;319(14):1444-1472. doi:10.1001/jama.2018.0158
3. Insel TR. Assessing the economic costs of serious mental illness. *Am J Psychiatry*. 2008;165(6):663-665. doi:10.1176/appi.ajp.2008.08030366
4. Florence CS, Zhou C, Luo F, Xu L. The Economic Burden of Prescription Opioid Overdose, Abuse, and Dependence in the United States, 2013. *Med Care*. 2016;54(10):901-906. doi:10.1097/MLR.0000000000000625
5. Kessler R, Chiu W. Prevalence, Severity, and Comorbidity of Twelve-month DSM-IV Disorders in the National Comorbidity Survey Replication (NCS- R). *Arch Gen …*. 2005;62(6):617-627. doi:10.1001/archpsyc.62.6.617.Prevalence
6. Grant BF, Goldstein RB, Saha TD, et al. Epidemiology of DSM-5 Alcohol Use Disorder: Results From the National Epidemiologic Survey on Alcohol and Related Conditions III. *JAMA Psychiatry*. 2015;72(8):757-766. doi:10.1001/jamapsychiatry.2015.0584
7. Grant BF, Saha TD, June Ruan W, et al. Epidemiology of DSM-5 drug use disorder results from the national epidemiologic survey on alcohol and related conditions-III. *JAMA Psychiatry*. 2016;73(1):39-47. doi:10.1001/jamapsychiatry.2015.2132
8. Hasin DS, Grant BF. The National Epidemiologic Survey on Alcohol and Related Conditions (NESARC) Waves 1 and 2: review and summary of findings. *Soc Psychiatry Psychiatr Epidemiol*. 2015;50(11):1609-1640. doi:10.1007/s00127-015-1088-0
9. Kessler RC, Merikangas KR. The National Comorbidity Survey Replication (NCS-R): Background and aims. *Int J Methods Psychiatr Res*. 2004;13(2):60-68. doi:10.1002/mpr.166
10. Demyttenaere K, Bruffaerts R, Posada-Villa J, et al. Prevalence, severity, and unmet need for treatment of mental disorders in the World Health Organization World Mental Health Surveys. *J Am Med Assoc*. 2004;291(21):2581-2590. doi:10.1001/jama.291.21.2581
11. Warden D, Rush AJ, Trivedi MH, Fava M, Wisniewski SR. The STAR*D project results: A comprehensive review of findings. *Curr Psychiatry Rep*. 2007;9(6):449-459. doi:10.1007/s11920-007-0061-3
12. Bycroft C, Freeman C, Petkova D, et al. The UK Biobank resource with deep phenotyping

and genomic data. *Nature*. 2018;562(7726):203-209. doi:10.1038/s41586-018-0579-z

13. Gaziano JM, Concato J, Brophy M, et al. Million Veteran Program: A mega-biobank to study genetic influences on health and disease. *J Clin Epidemiol*. 2016;70:214-223. doi:10.1016/j.jclinepi.2015.09.016
14. Nagai A, Hirata M, Kamatani Y, et al. Overview of the BioBank Japan Project: Study design and profile. *J Epidemiol*. 2017;27(3):S2-S8. doi:10.1016/j.je.2016.12.005
15. Mills MC, Rahal C. A scientometric review of genome-wide association studies. *Commun Biol*. 2019;2(1):9. doi:10.1038/s42003-018-0261-x
16. Martin AR, Kanai M, Kamatani Y, Okada Y, Neale BM, Daly MJ. Clinical use of current polygenic risk scores may exacerbate health disparities. *Nat Genet*. 2019;51(4):584-591. doi:10.1038/s41588-019-0379-x
17. The All of Us Research Program Investigators. The "All of Us" Research Program. *N Engl J Med*. 2019;381(7):668-676. doi:10.1056/nejmsr1809937
18. Zheutlin AB, Dennis J, Linnér RK, et al. Penetrance and pleiotropy of polygenic risk scores for schizophrenia in 106,160 patients across four health care systems. *Am J Psychiatry*. 2019;176(10):846-855. doi:10.1176/appi.ajp.2019.18091085
19. Denny JC, Bastarache L, Ritchie MD, et al. Systematic comparison of phenome-wide association study of electronic medical record data and genome-wide association study data. *Nat Biotechnol*. 2013;31(12):1102-1110. doi:10.1038/nbt.2749
20. Carroll RJ, Bastarache L, Denny JC. R PheWAS: Data analysis and plotting tools for phenome-wide association studies in the R environment. *Bioinformatics*. 2014;30(16):2375-2376. doi:10.1093/bioinformatics/btu197
21. Wei W-Q, Bastarache LA, Carroll RJ, et al. Evaluating phecodes, clinical classification software, and ICD-9-CM codes for phenome-wide association studies in the electronic health record. *PLoS One*. 2017;12(7):e0175508.
22. Wei WQ, Teixeira PL, Mo H, Cronin RM, Warner JL, Denny JC. Combining billing codes, clinical notes, and medications from electronic health records provides superior phenotyping performance. *J Am Med Informatics Assoc*. 2016;23(e1):20-27. doi:10.1093/jamia/ocv130
23. Davis KAS, Sudlow CLM, Hotopf M. Can mental health diagnoses in administrative data be used for research? A systematic review of the accuracy of routinely collected diagnoses. *BMC Psychiatry*. 2016;16(1):1-11. doi:10.1186/s12888-016-0963-x
24. Castro VM, Minnier J, Murphy SN, et al. Validation of electronic health record phenotyping of bipolar disorder cases and controls. *Am J Psychiatry*. 2015;172(4):363-372.

doi:10.1176/appi.ajp.2014.14030423

25. Sanchez-Roige S, Palmer AA, Fontanillas P, et al. Genome-wide association study meta-analysis of the alcohol use disorders identification test (AUDIT) in two population-based cohorts. *Am J Psychiatry*. 2019;176(2):107-118. doi:10.1176/appi.ajp.2018.18040369
26. Galmiche M, Déchelotte P, Lambert G, Tavolacci MP. Prevalence of eating disorders over the 2000-2018 period: A systematic literature review. *Am J Clin Nutr*. 2019;109(5):1402-1413. doi:10.1093/ajcn/nqy342
27. Trull TJ, Jahng S, Tomko RL, Wood PK, Sher KJ. Revised NESARC personality disorder diagnoses: Gender, prevalence, and comorbidity with substance dependence disorders. *J Pers Disord*. 2010;24(4):412-426. doi:10.1521/pedi.2010.24.4.412
28. Ohayon MM. Prevalence of DSM-IV diagnostic criteria of insomnia: Distinguishing insomnia related to mental disorders from sleep disorders. *J Psychiatr Res*. 1997;31(3):333-346. doi:10.1016/S0022-3956(97)00002-2
29. Perälä J, Suvisaari J, Saarni SI, et al. Lifetime prevalence of psychotic and bipolar I disorders in a general population. *Arch Gen Psychiatry*. 2007;64(1):19-28. doi:10.1001/archpsyc.64.1.19
30. Goldstein RB, Smith SM, Chou SP, et al. The epidemiology of DSM-5 posttraumatic stress disorder in the United States: results from the National Epidemiologic Survey on Alcohol and Related Conditions-III. *Soc Psychiatry Psychiatr Epidemiol*. 2016;51(8):1137-1148. doi:10.1007/s00127-016-1208-5
31. Kessler RC, Berglund P, Demler O, et al. Lifetime prevalence and age-of-onset distributions of DSM-IV disorders in the National Comorbidity Survey Replication. *Arch Gen Psychiatry*. 2005;62(6):593. doi:10.1001/archpsyc.62.6.593

© 2022 Barr PB et al. *JAMA Psychiatry*

6 ETABLE 1: ICD10CM CODES AND CORRESPONDING PHECODES

ICD10CM code	Phecode	ICD10CM description	Phecode description
F20	295	Schizophrenia	Schizophrenia and other psychotic disorders
F20	295	Schizophrenia	Schizophrenia and other psychotic disorders
F20.0	295	Paranoid schizophrenia	Schizophrenia and other psychotic disorders
F20.0	295	Paranoid schizophrenia	Schizophrenia and other psychotic disorders
F20.1	295	Disorganized schizophrenia	Schizophrenia and other psychotic disorders
F20.1	295	Disorganized schizophrenia	Schizophrenia and other psychotic disorders
F20.2	295	Catatonic schizophrenia	Schizophrenia and other psychotic disorders
F20.2	295	Catatonic schizophrenia	Schizophrenia and other psychotic disorders
F20.3	295	Undifferentiated schizophrenia	Schizophrenia and other psychotic disorders
F20.3	295	Undifferentiated schizophrenia	Schizophrenia and other psychotic disorders
F20.5	295	Residual schizophrenia	Schizophrenia and other psychotic disorders
F20.5	295	Residual schizophrenia	Schizophrenia and other psychotic disorders
F20.81	295	Schizophreniform disorder	Schizophrenia and other psychotic disorders
F20.81	295	Schizophreniform disorder	Schizophrenia and other psychotic disorders
F20.89	295	Other schizophrenia	Schizophrenia and other psychotic disorders
F20.89	295	Other schizophrenia	Schizophrenia and other psychotic disorders
F20.9	295	Schizophrenia, unspecified	Schizophrenia and other psychotic disorders
F20.9	295	Schizophrenia, unspecified	Schizophrenia and other psychotic disorders
F25	295	Schizoaffective disorders	Schizophrenia and other psychotic disorders
F25	295	Schizoaffective disorders	Schizophrenia and other psychotic disorders
F25.0	295	Schizoaffective disorder, bipolar type	Schizophrenia and other psychotic disorders
F25.0	295	Schizoaffective disorder, bipolar type	Schizophrenia and other psychotic disorders
F25.1	295	Schizoaffective disorder, depressive type	Schizophrenia and other psychotic disorders
F25.1	295	Schizoaffective disorder, depressive type	Schizophrenia and other psychotic disorders
F25.8	295	Other schizoaffective disorders	Schizophrenia and other psychotic disorders
F25.8	295	Other schizoaffective disorders	Schizophrenia and other psychotic disorders
F25.9	295	Schizoaffective disorder, unspecified	Schizophrenia and other psychotic disorders
F25.9	295	Schizoaffective disorder, unspecified	Schizophrenia and other psychotic disorders
F20	295.1	Schizophrenia	Schizophrenia
F20	295.1	Schizophrenia	Schizophrenia
F20.0	295.1	Paranoid schizophrenia	Schizophrenia
F20.0	295.1	Paranoid schizophrenia	Schizophrenia

© 2022 Barr PB et al. *JAMA Psychiatry*

F20.1	295.1	Disorganized schizophrenia	Schizophrenia
F20.1	295.1	Disorganized schizophrenia	Schizophrenia
F20.2	295.1	Catatonic schizophrenia	Schizophrenia
F20.2	295.1	Catatonic schizophrenia	Schizophrenia
F20.3	295.1	Undifferentiated schizophrenia	Schizophrenia
F20.3	295.1	Undifferentiated schizophrenia	Schizophrenia
F20.5	295.1	Residual schizophrenia	Schizophrenia
F20.5	295.1	Residual schizophrenia	Schizophrenia
F20.81	295.1	Schizophreniform disorder	Schizophrenia
F20.81	295.1	Schizophreniform disorder	Schizophrenia
F20.89	295.1	Other schizophrenia	Schizophrenia
F20.89	295.1	Other schizophrenia	Schizophrenia
F20.9	295.1	Schizophrenia, unspecified	Schizophrenia
F20.9	295.1	Schizophrenia, unspecified	Schizophrenia
F25	295.1	Schizoaffective disorders	Schizophrenia
F25	295.1	Schizoaffective disorders	Schizophrenia
F25.0	295.1	Schizoaffective disorder, bipolar type	Schizophrenia
F25.0	295.1	Schizoaffective disorder, bipolar type	Schizophrenia
F25.1	295.1	Schizoaffective disorder, depressive type	Schizophrenia
F25.1	295.1	Schizoaffective disorder, depressive type	Schizophrenia
F25.8	295.1	Other schizoaffective disorders	Schizophrenia
F25.8	295.1	Other schizoaffective disorders	Schizophrenia
F25.9	295.1	Schizoaffective disorder, unspecified	Schizophrenia
F25.9	295.1	Schizoaffective disorder, unspecified	Schizophrenia
F31	296	Bipolar disorder	Mood disorders
F31	296	Bipolar disorder	Mood disorders
F31.0	296	Bipolar disorder, current episode hypomanic	Mood disorders
F31.0	296	Bipolar disorder, current episode hypomanic	Mood disorders
F31.10	296	Bipolar disorder, current episode manic without psychotic features, unspecified	Mood disorders
F31.10	296	Bipolar disorder, current episode manic without psychotic features, unspecified	Mood disorders
F31.11	296	Bipolar disorder, current episode manic without psychotic features, mild	Mood disorders
F31.11	296	Bipolar disorder, current episode manic without psychotic features, mild	Mood disorders
F31.12	296	Bipolar disorder, current episode manic without psychotic features, moderate	Mood disorders
F31.12	296	Bipolar disorder, current episode manic without psychotic features, moderate	Mood disorders
F31.13	296	Bipolar disorder, current episode manic without psychotic features, severe	Mood disorders
F31.13	296	Bipolar disorder, current episode manic without psychotic features, severe	Mood disorders
F31.2	296	Bipolar disorder, current episode manic severe with psychotic features	Mood disorders
F31.2	296	Bipolar disorder, current episode manic severe with psychotic features	Mood disorders
F31.30	296	Bipolar disorder, current episode depressed, mild or moderate severity, unspecified	Mood disorders

© 2022 Barr PB et al. *JAMA Psychiatry*

F31.30	296	Bipolar disorder, current episode depressed, mild or moderate severity, unspecified	Mood disorders
F31.31	296	Bipolar disorder, current episode depressed, mild	Mood disorders
F31.31	296	Bipolar disorder, current episode depressed, mild	Mood disorders
F31.32	296	Bipolar disorder, current episode depressed, moderate	Mood disorders
F31.32	296	Bipolar disorder, current episode depressed, moderate	Mood disorders
F31.4	296	Bipolar disorder, current episode depressed, severe, without psychotic features	Mood disorders
F31.4	296	Bipolar disorder, current episode depressed, severe, without psychotic features	Mood disorders
F31.5	296	Bipolar disorder, current episode depressed, severe, with psychotic features	Mood disorders
F31.5	296	Bipolar disorder, current episode depressed, severe, with psychotic features	Mood disorders
F31.60	296	Bipolar disorder, current episode mixed, unspecified	Mood disorders
F31.60	296	Bipolar disorder, current episode mixed, unspecified	Mood disorders
F31.61	296	Bipolar disorder, current episode mixed, mild	Mood disorders
F31.61	296	Bipolar disorder, current episode mixed, mild	Mood disorders
F31.62	296	Bipolar disorder, current episode mixed, moderate	Mood disorders
F31.62	296	Bipolar disorder, current episode mixed, moderate	Mood disorders
F31.63	296	Bipolar disorder, current episode mixed, severe, without psychotic features	Mood disorders
F31.63	296	Bipolar disorder, current episode mixed, severe, without psychotic features	Mood disorders
F31.64	296	Bipolar disorder, current episode mixed, severe, with psychotic features	Mood disorders
F31.64	296	Bipolar disorder, current episode mixed, severe, with psychotic features	Mood disorders
F31.70	296	Bipolar disorder, currently in remission, most recent episode unspecified	Mood disorders
F31.70	296	Bipolar disorder, currently in remission, most recent episode unspecified	Mood disorders
F31.71	296	Bipolar disorder, in partial remission, most recent episode hypomanic	Mood disorders
F31.71	296	Bipolar disorder, in partial remission, most recent episode hypomanic	Mood disorders
F31.72	296	Bipolar disorder, in full remission, most recent episode hypomanic	Mood disorders
F31.72	296	Bipolar disorder, in full remission, most recent episode hypomanic	Mood disorders
F31.73	296	Bipolar disorder, in partial remission, most recent episode manic	Mood disorders
F31.73	296	Bipolar disorder, in partial remission, most recent episode manic	Mood disorders
F31.74	296	Bipolar disorder, in full remission, most recent episode manic	Mood disorders
F31.74	296	Bipolar disorder, in full remission, most recent episode manic	Mood disorders
F31.75	296	Bipolar disorder, in partial remission, most recent episode depressed	Mood disorders
F31.75	296	Bipolar disorder, in partial remission, most recent episode depressed	Mood disorders
F31.76	296	Bipolar disorder, in full remission, most recent episode depressed	Mood disorders
F31.76	296	Bipolar disorder, in full remission, most recent episode depressed	Mood disorders
F31.77	296	Bipolar disorder, in partial remission, most recent episode mixed	Mood disorders
F31.77	296	Bipolar disorder, in partial remission, most recent episode mixed	Mood disorders
F31.78	296	Bipolar disorder, in full remission, most recent episode mixed	Mood disorders
F31.78	296	Bipolar disorder, in full remission, most recent episode mixed	Mood disorders
F31.81	296	Bipolar II disorder	Mood disorders
F31.81	296	Bipolar II disorder	Mood disorders

F31.89	296	Other bipolar disorder	Mood disorders
F31.89	296	Other bipolar disorder	Mood disorders
F31.9	296	Bipolar disorder, unspecified	Mood disorders
F31.9	296	Bipolar disorder, unspecified	Mood disorders
F32	296	Major depressive disorder, single episode	Mood disorders
F32	296	Major depressive disorder, single episode	Mood disorders
F32.0	296	Major depressive disorder, single episode, mild	Mood disorders
F32.0	296	Major depressive disorder, single episode, mild	Mood disorders
F32.1	296	Major depressive disorder, single episode, moderate	Mood disorders
F32.1	296	Major depressive disorder, single episode, moderate	Mood disorders
F32.2	296	Major depressive disorder, single episode, severe without psychotic features	Mood disorders
F32.2	296	Major depressive disorder, single episode, severe without psychotic features	Mood disorders
F32.3	296	Major depressive disorder, single episode, severe with psychotic features	Mood disorders
F32.3	296	Major depressive disorder, single episode, severe with psychotic features	Mood disorders
F32.4	296	Major depressive disorder, single episode, in partial remission	Mood disorders
F32.4	296	Major depressive disorder, single episode, in partial remission	Mood disorders
F32.5	296	Major depressive disorder, single episode, in full remission	Mood disorders
F32.5	296	Major depressive disorder, single episode, in full remission	Mood disorders
F32.8	296	Other depressive episodes	Mood disorders
F32.8	296	Other depressive episodes	Mood disorders
F32.89	296	Other specified depressive episodes	Mood disorders
F32.89	296	Other specified depressive episodes	Mood disorders
F32.9	296	Major depressive disorder, single episode, unspecified	Mood disorders
F32.9	296	Major depressive disorder, single episode, unspecified	Mood disorders
F33	296	Major depressive disorder, recurrent	Mood disorders
F33	296	Major depressive disorder, recurrent	Mood disorders
F33.0	296	Major depressive disorder, recurrent, mild	Mood disorders
F33.0	296	Major depressive disorder, recurrent, mild	Mood disorders
F33.1	296	Major depressive disorder, recurrent, moderate	Mood disorders
F33.1	296	Major depressive disorder, recurrent, moderate	Mood disorders
F33.2	296	Major depressive disorder, recurrent severe without psychotic features	Mood disorders
F33.2	296	Major depressive disorder, recurrent severe without psychotic features	Mood disorders
F33.3	296	Major depressive disorder, recurrent, severe with psychotic symptoms	Mood disorders
F33.3	296	Major depressive disorder, recurrent, severe with psychotic symptoms	Mood disorders
F33.40	296	Major depressive disorder, recurrent, in remission, unspecified	Mood disorders
F33.40	296	Major depressive disorder, recurrent, in remission, unspecified	Mood disorders
F33.41	296	Major depressive disorder, recurrent, in partial remission	Mood disorders
F33.41	296	Major depressive disorder, recurrent, in partial remission	Mood disorders
F33.42	296	Major depressive disorder, recurrent, in full remission	Mood disorders

© 2022 Barr PB et al. *JAMA Psychiatry*

F33.42	296	Major depressive disorder, recurrent, in full remission	Mood disorders
F33.8	296	Other recurrent depressive disorders	Mood disorders
F33.8	296	Other recurrent depressive disorders	Mood disorders
F33.9	296	Major depressive disorder, recurrent, unspecified	Mood disorders
F33.9	296	Major depressive disorder, recurrent, unspecified	Mood disorders
F34.8	296	Other persistent mood [affective] disorders	Mood disorders
F34.8	296	Other persistent mood [affective] disorders	Mood disorders
F34.9	296	Persistent mood [affective] disorder, unspecified	Mood disorders
F34.9	296	Persistent mood [affective] disorder, unspecified	Mood disorders
F39	296	Unspecified mood [affective] disorder	Mood disorders
F39	296	Unspecified mood [affective] disorder	Mood disorders
F31	296.1	Bipolar disorder	Bipolar
F31	296.1	Bipolar disorder	Bipolar
F31.0	296.1	Bipolar disorder, current episode hypomanic	Bipolar
F31.0	296.1	Bipolar disorder, current episode hypomanic	Bipolar
F31.10	296.1	Bipolar disorder, current episode manic without psychotic features, unspecified	Bipolar
F31.10	296.1	Bipolar disorder, current episode manic without psychotic features, unspecified	Bipolar
F31.11	296.1	Bipolar disorder, current episode manic without psychotic features, mild	Bipolar
F31.11	296.1	Bipolar disorder, current episode manic without psychotic features, mild	Bipolar
F31.12	296.1	Bipolar disorder, current episode manic without psychotic features, moderate	Bipolar
F31.12	296.1	Bipolar disorder, current episode manic without psychotic features, moderate	Bipolar
F31.13	296.1	Bipolar disorder, current episode manic without psychotic features, severe	Bipolar
F31.13	296.1	Bipolar disorder, current episode manic without psychotic features, severe	Bipolar
F31.2	296.1	Bipolar disorder, current episode manic severe with psychotic features	Bipolar
F31.2	296.1	Bipolar disorder, current episode manic severe with psychotic features	Bipolar
F31.30	296.1	Bipolar disorder, current episode depressed, mild or moderate severity, unspecified	Bipolar
F31.30	296.1	Bipolar disorder, current episode depressed, mild or moderate severity, unspecified	Bipolar
F31.31	296.1	Bipolar disorder, current episode depressed, mild	Bipolar
F31.31	296.1	Bipolar disorder, current episode depressed, mild	Bipolar
F31.32	296.1	Bipolar disorder, current episode depressed, moderate	Bipolar
F31.32	296.1	Bipolar disorder, current episode depressed, moderate	Bipolar
F31.4	296.1	Bipolar disorder, current episode depressed, severe, without psychotic features	Bipolar
F31.4	296.1	Bipolar disorder, current episode depressed, severe, without psychotic features	Bipolar
F31.5	296.1	Bipolar disorder, current episode depressed, severe, with psychotic features	Bipolar
F31.5	296.1	Bipolar disorder, current episode depressed, severe, with psychotic features	Bipolar
F31.60	296.1	Bipolar disorder, current episode mixed, unspecified	Bipolar
F31.60	296.1	Bipolar disorder, current episode mixed, unspecified	Bipolar
F31.61	296.1	Bipolar disorder, current episode mixed, mild	Bipolar
F31.61	296.1	Bipolar disorder, current episode mixed, mild	Bipolar

© 2022 Barr PB et al. *JAMA Psychiatry*

F31.62	296.1	Bipolar disorder, current episode mixed, moderate	Bipolar
F31.62	296.1	Bipolar disorder, current episode mixed, moderate	Bipolar
F31.63	296.1	Bipolar disorder, current episode mixed, severe, without psychotic features	Bipolar
F31.63	296.1	Bipolar disorder, current episode mixed, severe, without psychotic features	Bipolar
F31.64	296.1	Bipolar disorder, current episode mixed, severe, with psychotic features	Bipolar
F31.64	296.1	Bipolar disorder, current episode mixed, severe, with psychotic features	Bipolar
F31.70	296.1	Bipolar disorder, currently in remission, most recent episode unspecified	Bipolar
F31.70	296.1	Bipolar disorder, currently in remission, most recent episode unspecified	Bipolar
F31.71	296.1	Bipolar disorder, in partial remission, most recent episode hypomanic	Bipolar
F31.71	296.1	Bipolar disorder, in partial remission, most recent episode hypomanic	Bipolar
F31.72	296.1	Bipolar disorder, in full remission, most recent episode hypomanic	Bipolar
F31.72	296.1	Bipolar disorder, in full remission, most recent episode hypomanic	Bipolar
F31.73	296.1	Bipolar disorder, in partial remission, most recent episode manic	Bipolar
F31.73	296.1	Bipolar disorder, in partial remission, most recent episode manic	Bipolar
F31.74	296.1	Bipolar disorder, in full remission, most recent episode manic	Bipolar
F31.74	296.1	Bipolar disorder, in full remission, most recent episode manic	Bipolar
F31.75	296.1	Bipolar disorder, in partial remission, most recent episode depressed	Bipolar
F31.75	296.1	Bipolar disorder, in partial remission, most recent episode depressed	Bipolar
F31.76	296.1	Bipolar disorder, in full remission, most recent episode depressed	Bipolar
F31.76	296.1	Bipolar disorder, in full remission, most recent episode depressed	Bipolar
F31.77	296.1	Bipolar disorder, in partial remission, most recent episode mixed	Bipolar
F31.77	296.1	Bipolar disorder, in partial remission, most recent episode mixed	Bipolar
F31.78	296.1	Bipolar disorder, in full remission, most recent episode mixed	Bipolar
F31.78	296.1	Bipolar disorder, in full remission, most recent episode mixed	Bipolar
F31.81	296.1	Bipolar II disorder	Bipolar
F31.81	296.1	Bipolar II disorder	Bipolar
F31.89	296.1	Other bipolar disorder	Bipolar
F31.89	296.1	Other bipolar disorder	Bipolar
F31.9	296.1	Bipolar disorder, unspecified	Bipolar
F31.9	296.1	Bipolar disorder, unspecified	Bipolar
F32.8	296.1	Other depressive episodes	Bipolar
F32.8	296.1	Other depressive episodes	Bipolar
F32.89	296.1	Other specified depressive episodes	Bipolar
F32.89	296.1	Other specified depressive episodes	Bipolar
F32	296.2	Major depressive disorder, single episode	Depression
F32	296.2	Major depressive disorder, single episode	Depression
F32.0	296.2	Major depressive disorder, single episode, mild	Depression
F32.0	296.2	Major depressive disorder, single episode, mild	Depression
F32.1	296.2	Major depressive disorder, single episode, moderate	Depression

F32.1	296.2	Major depressive disorder, single episode, moderate	Depression
F32.2	296.2	Major depressive disorder, single episode, severe without psychotic features	Depression
F32.2	296.2	Major depressive disorder, single episode, severe without psychotic features	Depression
F32.3	296.2	Major depressive disorder, single episode, severe with psychotic features	Depression
F32.3	296.2	Major depressive disorder, single episode, severe with psychotic features	Depression
F32.4	296.2	Major depressive disorder, single episode, in partial remission	Depression
F32.4	296.2	Major depressive disorder, single episode, in partial remission	Depression
F32.5	296.2	Major depressive disorder, single episode, in full remission	Depression
F32.5	296.2	Major depressive disorder, single episode, in full remission	Depression
F32.9	296.2	Major depressive disorder, single episode, unspecified	Depression
F32.9	296.2	Major depressive disorder, single episode, unspecified	Depression
F33	296.2	Major depressive disorder, recurrent	Depression
F33	296.2	Major depressive disorder, recurrent	Depression
F33.0	296.2	Major depressive disorder, recurrent, mild	Depression
F33.0	296.2	Major depressive disorder, recurrent, mild	Depression
F33.1	296.2	Major depressive disorder, recurrent, moderate	Depression
F33.1	296.2	Major depressive disorder, recurrent, moderate	Depression
F33.2	296.2	Major depressive disorder, recurrent severe without psychotic features	Depression
F33.2	296.2	Major depressive disorder, recurrent severe without psychotic features	Depression
F33.3	296.2	Major depressive disorder, recurrent, severe with psychotic symptoms	Depression
F33.3	296.2	Major depressive disorder, recurrent, severe with psychotic symptoms	Depression
F33.40	296.2	Major depressive disorder, recurrent, in remission, unspecified	Depression
F33.40	296.2	Major depressive disorder, recurrent, in remission, unspecified	Depression
F33.41	296.2	Major depressive disorder, recurrent, in partial remission	Depression
F33.41	296.2	Major depressive disorder, recurrent, in partial remission	Depression
F33.42	296.2	Major depressive disorder, recurrent, in full remission	Depression
F33.42	296.2	Major depressive disorder, recurrent, in full remission	Depression
F33.9	296.2	Major depressive disorder, recurrent, unspecified	Depression
F33.9	296.2	Major depressive disorder, recurrent, unspecified	Depression
F32	296.22	Major depressive disorder, single episode	Major depressive disorder
F32	296.22	Major depressive disorder, single episode	Major depressive disorder
F32.1	296.22	Major depressive disorder, single episode, moderate	Major depressive disorder
F32.1	296.22	Major depressive disorder, single episode, moderate	Major depressive disorder
F32.2	296.22	Major depressive disorder, single episode, severe without psychotic features	Major depressive disorder
F32.2	296.22	Major depressive disorder, single episode, severe without psychotic features	Major depressive disorder
F32.3	296.22	Major depressive disorder, single episode, severe with psychotic features	Major depressive disorder
F32.3	296.22	Major depressive disorder, single episode, severe with psychotic features	Major depressive disorder
F32.4	296.22	Major depressive disorder, single episode, in partial remission	Major depressive disorder

© 2022 Barr PB et al. *JAMA Psychiatry*

F32.4	296.2 2	Major depressive disorder, single episode, in partial remission	Major depressive disorder
F32.5	296.2 2	Major depressive disorder, single episode, in full remission	Major depressive disorder
F32.5	296.2 2	Major depressive disorder, single episode, in full remission	Major depressive disorder
F32.9	296.2 2	Major depressive disorder, single episode, unspecified	Major depressive disorder
F32.9	296.2 2	Major depressive disorder, single episode, unspecified	Major depressive disorder
F33	296.2 2	Major depressive disorder, recurrent	Major depressive disorder
F33	296.2 2	Major depressive disorder, recurrent	Major depressive disorder
F33.1	296.2 2	Major depressive disorder, recurrent, moderate	Major depressive disorder
F33.1	296.2 2	Major depressive disorder, recurrent, moderate	Major depressive disorder
F33.2	296.2 2	Major depressive disorder, recurrent severe without psychotic features	Major depressive disorder
F33.2	296.2 2	Major depressive disorder, recurrent severe without psychotic features	Major depressive disorder
F33.3	296.2 2	Major depressive disorder, recurrent, severe with psychotic symptoms	Major depressive disorder
F33.3	296.2 2	Major depressive disorder, recurrent, severe with psychotic symptoms	Major depressive disorder
F33.40	296.2 2	Major depressive disorder, recurrent, in remission, unspecified	Major depressive disorder
F33.40	296.2 2	Major depressive disorder, recurrent, in remission, unspecified	Major depressive disorder
F33.41	296.2 2	Major depressive disorder, recurrent, in partial remission	Major depressive disorder
F33.41	296.2 2	Major depressive disorder, recurrent, in partial remission	Major depressive disorder
F33.42	296.2 2	Major depressive disorder, recurrent, in full remission	Major depressive disorder
F33.42	296.2 2	Major depressive disorder, recurrent, in full remission	Major depressive disorder
F33.9	296.2 2	Major depressive disorder, recurrent, unspecified	Major depressive disorder
F33.9	296.2 2	Major depressive disorder, recurrent, unspecified	Major depressive disorder
F34.1	300	Dysthymic disorder	Anxiety disorders
F34.1	300	Dysthymic disorder	Anxiety disorders
F40.00	300	Agoraphobia, unspecified	Anxiety disorders
F40.00	300	Agoraphobia, unspecified	Anxiety disorders
F40.01	300	Agoraphobia with panic disorder	Anxiety disorders
F40.01	300	Agoraphobia with panic disorder	Anxiety disorders
F40.02	300	Agoraphobia without panic disorder	Anxiety disorders
F40.02	300	Agoraphobia without panic disorder	Anxiety disorders
F40.10	300	Social phobia, unspecified	Anxiety disorders
F40.10	300	Social phobia, unspecified	Anxiety disorders
F40.11	300	Social phobia, generalized	Anxiety disorders
F40.11	300	Social phobia, generalized	Anxiety disorders
F40.210	300	Arachnophobia	Anxiety disorders
F40.210	300	Arachnophobia	Anxiety disorders
F40.218	300	Other animal type phobia	Anxiety disorders
F40.218	300	Other animal type phobia	Anxiety disorders

F40.231	300	Fear of injections and transfusions	Anxiety disorders
F40.231	300	Fear of injections and transfusions	Anxiety disorders
F40.232	300	Fear of other medical care	Anxiety disorders
F40.232	300	Fear of other medical care	Anxiety disorders
F40.233	300	Fear of injury	Anxiety disorders
F40.233	300	Fear of injury	Anxiety disorders
F40.240	300	Claustrophobia	Anxiety disorders
F40.240	300	Claustrophobia	Anxiety disorders
F40.241	300	Acrophobia	Anxiety disorders
F40.241	300	Acrophobia	Anxiety disorders
F40.242	300	Fear of bridges	Anxiety disorders
F40.242	300	Fear of bridges	Anxiety disorders
F40.243	300	Fear of flying	Anxiety disorders
F40.243	300	Fear of flying	Anxiety disorders
F40.248	300	Other situational type phobia	Anxiety disorders
F40.248	300	Other situational type phobia	Anxiety disorders
F40.291	300	Gynephobia	Anxiety disorders
F40.291	300	Gynephobia	Anxiety disorders
F40.298	300	Other specified phobia	Anxiety disorders
F40.298	300	Other specified phobia	Anxiety disorders
F40.8	300	Other phobic anxiety disorders	Anxiety disorders
F40.8	300	Other phobic anxiety disorders	Anxiety disorders
F40.9	300	Phobic anxiety disorder, unspecified	Anxiety disorders
F40.9	300	Phobic anxiety disorder, unspecified	Anxiety disorders
F41.0	300	Panic disorder [episodic paroxysmal anxiety]	Anxiety disorders
F41.0	300	Panic disorder [episodic paroxysmal anxiety]	Anxiety disorders
F41.1	300	Generalized anxiety disorder	Anxiety disorders
F41.1	300	Generalized anxiety disorder	Anxiety disorders
F41.3	300	Other mixed anxiety disorders	Anxiety disorders
F41.3	300	Other mixed anxiety disorders	Anxiety disorders
F41.8	300	Other specified anxiety disorders	Anxiety disorders
F41.8	300	Other specified anxiety disorders	Anxiety disorders
F41.9	300	Anxiety disorder, unspecified	Anxiety disorders
F41.9	300	Anxiety disorder, unspecified	Anxiety disorders
F42	300	Obsessive-compulsive disorder	Anxiety disorders
F42	300	Obsessive-compulsive disorder	Anxiety disorders
F42.8	300	Other obsessive-compulsive disorder	Anxiety disorders
F42.8	300	Other obsessive-compulsive disorder	Anxiety disorders
F42.9	300	Obsessive-compulsive disorder, unspecified	Anxiety disorders

© 2022 Barr PB et al. *JAMA Psychiatry*

F42.9	300	Obsessive-compulsive disorder, unspecified	Anxiety disorders
F43.1	300	Post-traumatic stress disorder (PTSD)	Anxiety disorders
F43.1	300	Post-traumatic stress disorder (PTSD)	Anxiety disorders
F43.10	300	Post-traumatic stress disorder, unspecified	Anxiety disorders
F43.10	300	Post-traumatic stress disorder, unspecified	Anxiety disorders
F43.11	300	Post-traumatic stress disorder, acute	Anxiety disorders
F43.11	300	Post-traumatic stress disorder, acute	Anxiety disorders
F43.12	300	Post-traumatic stress disorder, chronic	Anxiety disorders
F43.12	300	Post-traumatic stress disorder, chronic	Anxiety disorders
F40.00	300.1	Agoraphobia, unspecified	Anxiety disorder
F40.00	300.1	Agoraphobia, unspecified	Anxiety disorder
F40.01	300.1	Agoraphobia with panic disorder	Anxiety disorder
F40.01	300.1	Agoraphobia with panic disorder	Anxiety disorder
F40.02	300.1	Agoraphobia without panic disorder	Anxiety disorder
F40.02	300.1	Agoraphobia without panic disorder	Anxiety disorder
F40.10	300.1	Social phobia, unspecified	Anxiety disorder
F40.10	300.1	Social phobia, unspecified	Anxiety disorder
F40.11	300.1	Social phobia, generalized	Anxiety disorder
F40.11	300.1	Social phobia, generalized	Anxiety disorder
F40.210	300.1	Arachnophobia	Anxiety disorder
F40.210	300.1	Arachnophobia	Anxiety disorder
F40.218	300.1	Other animal type phobia	Anxiety disorder
F40.218	300.1	Other animal type phobia	Anxiety disorder
F40.231	300.1	Fear of injections and transfusions	Anxiety disorder
F40.231	300.1	Fear of injections and transfusions	Anxiety disorder
F40.232	300.1	Fear of other medical care	Anxiety disorder
F40.232	300.1	Fear of other medical care	Anxiety disorder
F40.233	300.1	Fear of injury	Anxiety disorder
F40.233	300.1	Fear of injury	Anxiety disorder
F40.240	300.1	Claustrophobia	Anxiety disorder
F40.240	300.1	Claustrophobia	Anxiety disorder
F40.241	300.1	Acrophobia	Anxiety disorder
F40.241	300.1	Acrophobia	Anxiety disorder
F40.242	300.1	Fear of bridges	Anxiety disorder
F40.242	300.1	Fear of bridges	Anxiety disorder
F40.243	300.1	Fear of flying	Anxiety disorder
F40.243	300.1	Fear of flying	Anxiety disorder
F40.248	300.1	Other situational type phobia	Anxiety disorder
F40.248	300.1	Other situational type phobia	Anxiety disorder

© 2022 Barr PB et al. *JAMA Psychiatry*

F40.291	300.1	Gynephobia	Anxiety disorder
F40.291	300.1	Gynephobia	Anxiety disorder
F40.298	300.1	Other specified phobia	Anxiety disorder
F40.298	300.1	Other specified phobia	Anxiety disorder
F40.8	300.1	Other phobic anxiety disorders	Anxiety disorder
F40.8	300.1	Other phobic anxiety disorders	Anxiety disorder
F40.9	300.1	Phobic anxiety disorder, unspecified	Anxiety disorder
F40.9	300.1	Phobic anxiety disorder, unspecified	Anxiety disorder
F41.0	300.1	Panic disorder [episodic paroxysmal anxiety]	Anxiety disorder
F41.0	300.1	Panic disorder [episodic paroxysmal anxiety]	Anxiety disorder
F41.1	300.1	Generalized anxiety disorder	Anxiety disorder
F41.1	300.1	Generalized anxiety disorder	Anxiety disorder
F41.3	300.1	Other mixed anxiety disorders	Anxiety disorder
F41.3	300.1	Other mixed anxiety disorders	Anxiety disorder
F41.8	300.1	Other specified anxiety disorders	Anxiety disorder
F41.8	300.1	Other specified anxiety disorders	Anxiety disorder
F41.9	300.1	Anxiety disorder, unspecified	Anxiety disorder
F41.9	300.1	Anxiety disorder, unspecified	Anxiety disorder
F41.1	300.1<br>1	Generalized anxiety disorder	Generalized anxiety disorder
F41.1	300.1<br>1	Generalized anxiety disorder	Generalized anxiety disorder
F40.00	300.1<br>2	Agoraphobia, unspecified	Agoraphobia, social phobia, and panic disorder
F40.00	300.1<br>2	Agoraphobia, unspecified	Agoraphobia, social phobia, and panic disorder
F40.01	300.1<br>2	Agoraphobia with panic disorder	Agoraphobia, social phobia, and panic disorder
F40.01	300.1<br>2	Agoraphobia with panic disorder	Agoraphobia, social phobia, and panic disorder
F40.02	300.1<br>2	Agoraphobia without panic disorder	Agoraphobia, social phobia, and panic disorder
F40.02	300.1<br>2	Agoraphobia without panic disorder	Agoraphobia, social phobia, and panic disorder
F40.10	300.1<br>2	Social phobia, unspecified	Agoraphobia, social phobia, and panic disorder
F40.10	300.1<br>2	Social phobia, unspecified	Agoraphobia, social phobia, and panic disorder
F40.11	300.1<br>2	Social phobia, generalized	Agoraphobia, social phobia, and panic disorder
F40.11	300.1<br>2	Social phobia, generalized	Agoraphobia, social phobia, and panic disorder
F41.0	300.1<br>2	Panic disorder [episodic paroxysmal anxiety]	Agoraphobia, social phobia, and panic disorder
F41.0	300.1<br>2	Panic disorder [episodic paroxysmal anxiety]	Agoraphobia, social phobia, and panic disorder
F40.210	300.1<br>3	Arachnophobia	Phobia
F40.210	300.1<br>3	Arachnophobia	Phobia
F40.218	300.1<br>3	Other animal type phobia	Phobia
F40.218	300.1<br>3	Other animal type phobia	Phobia
F40.231	300.1<br>3	Fear of injections and transfusions	Phobia

© 2022 Barr PB et al. *JAMA Psychiatry*

F40.231	300.13	Fear of injections and transfusions	Phobia
F40.232	300.13	Fear of other medical care	Phobia
F40.232	300.13	Fear of other medical care	Phobia
F40.233	300.13	Fear of injury	Phobia
F40.233	300.13	Fear of injury	Phobia
F40.240	300.13	Claustrophobia	Phobia
F40.240	300.13	Claustrophobia	Phobia
F40.241	300.13	Acrophobia	Phobia
F40.241	300.13	Acrophobia	Phobia
F40.242	300.13	Fear of bridges	Phobia
F40.242	300.13	Fear of bridges	Phobia
F40.243	300.13	Fear of flying	Phobia
F40.243	300.13	Fear of flying	Phobia
F40.248	300.13	Other situational type phobia	Phobia
F40.248	300.13	Other situational type phobia	Phobia
F40.291	300.13	Gynephobia	Phobia
F40.291	300.13	Gynephobia	Phobia
F40.298	300.13	Other specified phobia	Phobia
F40.298	300.13	Other specified phobia	Phobia
F40.8	300.13	Other phobic anxiety disorders	Phobia
F40.8	300.13	Other phobic anxiety disorders	Phobia
F40.9	300.13	Phobic anxiety disorder, unspecified	Phobia
F40.9	300.13	Phobic anxiety disorder, unspecified	Phobia
F42	300.3	Obsessive-compulsive disorder	Obsessive-compulsive disorders
F42	300.3	Obsessive-compulsive disorder	Obsessive-compulsive disorders
F42.8	300.3	Other obsessive-compulsive disorder	Obsessive-compulsive disorders
F42.8	300.3	Other obsessive-compulsive disorder	Obsessive-compulsive disorders
F42.9	300.3	Obsessive-compulsive disorder, unspecified	Obsessive-compulsive disorders
F42.9	300.3	Obsessive-compulsive disorder, unspecified	Obsessive-compulsive disorders
F34.1	300.4	Dysthymic disorder	Dysthymic disorder
F34.1	300.4	Dysthymic disorder	Dysthymic disorder
F43.1	300.9	Post-traumatic stress disorder (PTSD)	Posttraumatic stress disorder
F43.1	300.9	Post-traumatic stress disorder (PTSD)	Posttraumatic stress disorder
F43.10	300.9	Post-traumatic stress disorder, unspecified	Posttraumatic stress disorder
F43.10	300.9	Post-traumatic stress disorder, unspecified	Posttraumatic stress disorder
F43.11	300.9	Post-traumatic stress disorder, acute	Posttraumatic stress disorder
F43.11	300.9	Post-traumatic stress disorder, acute	Posttraumatic stress disorder

© 2022 Barr PB et al. *JAMA Psychiatry*

F43.12	300.9	Post-traumatic stress disorder, chronic	Posttraumatic stress disorder
F43.12	300.9	Post-traumatic stress disorder, chronic	Posttraumatic stress disorder
F34.0	301	Cyclothymic disorder	Personality disorders
F34.0	301	Cyclothymic disorder	Personality disorders
F60.0	301	Paranoid personality disorder	Personality disorders
F60.0	301	Paranoid personality disorder	Personality disorders
F60.1	301	Schizoid personality disorder	Personality disorders
F60.1	301	Schizoid personality disorder	Personality disorders
F60.2	301	Antisocial personality disorder	Personality disorders
F60.2	301	Antisocial personality disorder	Personality disorders
F60.3	301	Borderline personality disorder	Personality disorders
F60.3	301	Borderline personality disorder	Personality disorders
F60.4	301	Histrionic personality disorder	Personality disorders
F60.4	301	Histrionic personality disorder	Personality disorders
F60.5	301	Obsessive-compulsive personality disorder	Personality disorders
F60.5	301	Obsessive-compulsive personality disorder	Personality disorders
F60.6	301	Avoidant personality disorder	Personality disorders
F60.6	301	Avoidant personality disorder	Personality disorders
F60.7	301	Dependent personality disorder	Personality disorders
F60.7	301	Dependent personality disorder	Personality disorders
F60.81	301	Narcissistic personality disorder	Personality disorders
F60.81	301	Narcissistic personality disorder	Personality disorders
F60.89	301	Other specific personality disorders	Personality disorders
F60.89	301	Other specific personality disorders	Personality disorders
F60.9	301	Personality disorder, unspecified	Personality disorders
F60.9	301	Personality disorder, unspecified	Personality disorders
F60.1	301.1	Schizoid personality disorder	Schizoid personality disorder
F60.1	301.1	Schizoid personality disorder	Schizoid personality disorder
F60.5	301.1	Obsessive-compulsive personality disorder	Schizoid personality disorder
F60.5	301.1	Obsessive-compulsive personality disorder	Schizoid personality disorder
F60.2	301.2	Antisocial personality disorder	Antisocial/borderline personality disorder
F60.2	301.2	Antisocial personality disorder	Antisocial/borderline personality disorder
F60.3	301.2	Borderline personality disorder	Antisocial/borderline personality disorder
F60.3	301.2	Borderline personality disorder	Antisocial/borderline personality disorder
F43.20	304	Adjustment disorder, unspecified	Adjustment reaction
F43.20	304	Adjustment disorder, unspecified	Adjustment reaction
F43.21	304	Adjustment disorder with depressed mood	Adjustment reaction
F43.21	304	Adjustment disorder with depressed mood	Adjustment reaction

© 2022 Barr PB et al. *JAMA Psychiatry*

F43.22	304	Adjustment disorder with anxiety	Adjustment reaction
F43.22	304	Adjustment disorder with anxiety	Adjustment reaction
F43.23	304	Adjustment disorder with mixed anxiety and depressed mood	Adjustment reaction
F43.23	304	Adjustment disorder with mixed anxiety and depressed mood	Adjustment reaction
F43.24	304	Adjustment disorder with disturbance of conduct	Adjustment reaction
F43.25	304	Adjustment disorder with mixed disturbance of emotions and conduct	Adjustment reaction
F43.29	304	Adjustment disorder with other symptoms	Adjustment reaction
F50.00	305.2	Anorexia nervosa, unspecified	Eating disorder
F50.01	305.2	Anorexia nervosa, restricting type	Eating disorder
F50.02	305.2	Anorexia nervosa, binge eating/purging type	Eating disorder
F50.2	305.2	Bulimia nervosa	Eating disorder
F50.8	305.2	Other eating disorders	Eating disorder
F50.89	305.2	Other specified eating disorder	Eating disorder
F50.9	305.2	Eating disorder, unspecified	Eating disorder
F50.00	305.21	Anorexia nervosa, unspecified	Anorexia nervosa
F50.01	305.21	Anorexia nervosa, restricting type	Anorexia nervosa
F50.02	305.21	Anorexia nervosa, binge eating/purging type	Anorexia nervosa
F91.1	312	Conduct disorder, childhood-onset type	Conduct disorders
F91.2	312	Conduct disorder, adolescent-onset type	Conduct disorders
F91.3	312	Oppositional defiant disorder	Conduct disorders
F91.8	312	Other conduct disorders	Conduct disorders
F91.9	312	Conduct disorder, unspecified	Conduct disorders
F90.0	313	Attention-deficit hyperactivity disorder, predominantly inattentive type	Pervasive developmental disorders
F90.1	313	Attention-deficit hyperactivity disorder, predominantly hyperactive type	Pervasive developmental disorders
F90.2	313	Attention-deficit hyperactivity disorder, combined type	Pervasive developmental disorders
F90.8	313	Attention-deficit hyperactivity disorder, other type	Pervasive developmental disorders
F90.9	313	Attention-deficit hyperactivity disorder, unspecified type	Pervasive developmental disorders
F90.0	313.1	Attention-deficit hyperactivity disorder, predominantly inattentive type	Attention deficit hyperactivity disorder
F90.1	313.1	Attention-deficit hyperactivity disorder, predominantly hyperactive type	Attention deficit hyperactivity disorder
F90.2	313.1	Attention-deficit hyperactivity disorder, combined type	Attention deficit hyperactivity disorder
F90.8	313.1	Attention-deficit hyperactivity disorder, other type	Attention deficit hyperactivity disorder
F90.9	313.1	Attention-deficit hyperactivity disorder, unspecified type	Attention deficit hyperactivity disorder
F11.1	316	Opioid abuse	Substance addiction and disorders
F11.10	316	Opioid abuse, uncomplicated	Substance addiction and disorders
F11.120	316	Opioid abuse with intoxication, uncomplicated	Substance addiction and disorders
F11.122	316	Opioid abuse with intoxication with perceptual disturbance	Substance addiction and disorders
F11.129	316	Opioid abuse with intoxication, unspecified	Substance addiction and disorders

© 2022 Barr PB et al. *JAMA Psychiatry*

F11.14	316	Opioid abuse with opioid-induced mood disorder	Substance addiction and disorders
F11.151	316	Opioid abuse with opioid-induced psychotic disorder with hallucinations	Substance addiction and disorders
F11.159	316	Opioid abuse with opioid-induced psychotic disorder, unspecified	Substance addiction and disorders
F11.182	316	Opioid abuse with opioid-induced sleep disorder	Substance addiction and disorders
F11.188	316	Opioid abuse with other opioid-induced disorder	Substance addiction and disorders
F11.19	316	Opioid abuse with unspecified opioid-induced disorder	Substance addiction and disorders
F11.2	316	Opioid dependence	Substance addiction and disorders
F11.20	316	Opioid dependence, uncomplicated	Substance addiction and disorders
F11.21	316	Opioid dependence, in remission	Substance addiction and disorders
F11.220	316	Opioid dependence with intoxication, uncomplicated	Substance addiction and disorders
F11.221	316	Opioid dependence with intoxication delirium	Substance addiction and disorders
F11.222	316	Opioid dependence with intoxication with perceptual disturbance	Substance addiction and disorders
F11.229	316	Opioid dependence with intoxication, unspecified	Substance addiction and disorders
F11.23	316	Opioid dependence with withdrawal	Substance addiction and disorders
F11.24	316	Opioid dependence with opioid-induced mood disorder	Substance addiction and disorders
F11.250	316	Opioid dependence with opioid-induced psychotic disorder with delusions	Substance addiction and disorders
F11.251	316	Opioid dependence with opioid-induced psychotic disorder with hallucinations	Substance addiction and disorders
F11.259	316	Opioid dependence with opioid-induced psychotic disorder, unspecified	Substance addiction and disorders
F11.281	316	Opioid dependence with opioid-induced sexual dysfunction	Substance addiction and disorders
F11.282	316	Opioid dependence with opioid-induced sleep disorder	Substance addiction and disorders
F11.288	316	Opioid dependence with other opioid-induced disorder	Substance addiction and disorders
F11.29	316	Opioid dependence with unspecified opioid-induced disorder	Substance addiction and disorders
F12.1	316	Cannabis abuse	Substance addiction and disorders
F12.10	316	Cannabis abuse, uncomplicated	Substance addiction and disorders
F12.120	316	Cannabis abuse with intoxication, uncomplicated	Substance addiction and disorders
F12.122	316	Cannabis abuse with intoxication with perceptual disturbance	Substance addiction and disorders
F12.129	316	Cannabis abuse with intoxication, unspecified	Substance addiction and disorders
F12.150	316	Cannabis abuse with psychotic disorder with delusions	Substance addiction and disorders
F12.151	316	Cannabis abuse with psychotic disorder with hallucinations	Substance addiction and disorders
F12.159	316	Cannabis abuse with psychotic disorder, unspecified	Substance addiction and disorders
F12.180	316	Cannabis abuse with cannabis-induced anxiety disorder	Substance addiction and disorders
F12.188	316	Cannabis abuse with other cannabis-induced disorder	Substance addiction and disorders
F12.19	316	Cannabis abuse with unspecified cannabis-induced disorder	Substance addiction and disorders
F12.2	316	Cannabis dependence	Substance addiction and disorders
F12.20	316	Cannabis dependence, uncomplicated	Substance addiction and disorders
F12.21	316	Cannabis dependence, in remission	Substance addiction and disorders

© 2022 Barr PB et al. *JAMA Psychiatry*

F12.220	316	Cannabis dependence with intoxication, uncomplicated	Substance addiction and disorders
F12.229	316	Cannabis dependence with intoxication, unspecified	Substance addiction and disorders
F12.259	316	Cannabis dependence with psychotic disorder, unspecified	Substance addiction and disorders
F12.280	316	Cannabis dependence with cannabis-induced anxiety disorder	Substance addiction and disorders
F12.288	316	Cannabis dependence with other cannabis-induced disorder	Substance addiction and disorders
F12.29	316	Cannabis dependence with unspecified cannabis-induced disorder	Substance addiction and disorders
F13.10	316	Sedative, hypnotic, or anxiolytic abuse, uncomplicated	Substance addiction and disorders
F13.120	316	Sedative, hypnotic, or anxiolytic abuse with intoxication, uncomplicated	Substance addiction and disorders
F13.129	316	Sedative, hypnotic, or anxiolytic abuse with intoxication, unspecified	Substance addiction and disorders
F13.14	316	Sedative, hypnotic or anxiolytic abuse with sedative, hypnotic, or anxiolytic-induced mood disorder	Substance addiction and disorders
F13.150	316	Sedative, hypnotic or anxiolytic abuse with sedative, hypnotic, or anxiolytic-induced psychotic disorder with delusions	Substance addiction and disorders
F13.151	316	Sedative, hypnotic or anxiolytic abuse with sedative, hypnotic, or anxiolytic-induced psychotic disorder with hallucinations	Substance addiction and disorders
F13.159	316	Sedative, hypnotic or anxiolytic abuse with sedative, hypnotic, or anxiolytic-induced psychotic disorder, unspecified	Substance addiction and disorders
F13.180	316	Sedative, hypnotic or anxiolytic abuse with sedative, hypnotic, or anxiolytic-induced anxiety disorder	Substance addiction and disorders
F13.188	316	Sedative, hypnotic or anxiolytic abuse with other sedative, hypnotic, or anxiolytic-induced disorder	Substance addiction and disorders
F13.19	316	Sedative, hypnotic or anxiolytic abuse with unspecified sedative, hypnotic, or anxiolytic-induced disorder	Substance addiction and disorders
F13.20	316	Sedative, hypnotic, or anxiolytic dependence, uncomplicated	Substance addiction and disorders
F13.21	316	Sedative, hypnotic, or anxiolytic dependence, in remission	Substance addiction and disorders
F13.220	316	Sedative, hypnotic, or anxiolytic dependence with intoxication, uncomplicated	Substance addiction and disorders
F13.221	316	Sedative, hypnotic, or anxiolytic dependence with intoxication delirium	Substance addiction and disorders
F13.229	316	Sedative, hypnotic, or anxiolytic dependence with intoxication, unspecified	Substance addiction and disorders
F13.230	316	Sedative, hypnotic, or anxiolytic dependence with withdrawal, uncomplicated	Substance addiction and disorders
F13.231	316	Sedative, hypnotic, or anxiolytic dependence with withdrawal delirium	Substance addiction and disorders
F13.232	316	Sedative, hypnotic, or anxiolytic dependence with withdrawal with perceptual disturbance	Substance addiction and disorders
F13.239	316	Sedative, hypnotic, or anxiolytic dependence with withdrawal, unspecified	Substance addiction and disorders
F13.24	316	Sedative, hypnotic or anxiolytic dependence with sedative, hypnotic, or anxiolytic-induced mood disorder	Substance addiction and disorders
F13.280	316	Sedative, hypnotic or anxiolytic dependence with sedative, hypnotic, or anxiolytic-induced anxiety disorder	Substance addiction and disorders
F13.282	316	Sedative, hypnotic or anxiolytic dependence with sedative, hypnotic, or anxiolytic-induced sleep disorder	Substance addiction and disorders
F13.288	316	Sedative, hypnotic or anxiolytic dependence with other sedative, hypnotic, or anxiolytic-induced disorder	Substance addiction and disorders
F13.29	316	Sedative, hypnotic or anxiolytic dependence with unspecified sedative, hypnotic, or anxiolytic-induced disorder	Substance addiction and disorders
F14.1	316	Cocaine abuse	Substance addiction and disorders
F14.10	316	Cocaine abuse, uncomplicated	Substance addiction and disorders
F14.120	316	Cocaine abuse with intoxication, uncomplicated	Substance addiction and disorders
F14.122	316	Cocaine abuse with intoxication with perceptual disturbance	Substance addiction and disorders
F14.129	316	Cocaine abuse with intoxication, unspecified	Substance addiction and disorders
F14.14	316	Cocaine abuse with cocaine-induced mood disorder	Substance addiction and disorders

© 2022 Barr PB et al. *JAMA Psychiatry*

F14.150	316	Cocaine abuse with cocaine-induced psychotic disorder with delusions	Substance addiction and disorders
F14.151	316	Cocaine abuse with cocaine-induced psychotic disorder with hallucinations	Substance addiction and disorders
F14.159	316	Cocaine abuse with cocaine-induced psychotic disorder, unspecified	Substance addiction and disorders
F14.180	316	Cocaine abuse with cocaine-induced anxiety disorder	Substance addiction and disorders
F14.181	316	Cocaine abuse with cocaine-induced sexual dysfunction	Substance addiction and disorders
F14.182	316	Cocaine abuse with cocaine-induced sleep disorder	Substance addiction and disorders
F14.188	316	Cocaine abuse with other cocaine-induced disorder	Substance addiction and disorders
F14.19	316	Cocaine abuse with unspecified cocaine-induced disorder	Substance addiction and disorders
F14.20	316	Cocaine dependence, uncomplicated	Substance addiction and disorders
F14.21	316	Cocaine dependence, in remission	Substance addiction and disorders
F14.220	316	Cocaine dependence with intoxication, uncomplicated	Substance addiction and disorders
F14.221	316	Cocaine dependence with intoxication delirium	Substance addiction and disorders
F14.222	316	Cocaine dependence with intoxication with perceptual disturbance	Substance addiction and disorders
F14.229	316	Cocaine dependence with intoxication, unspecified	Substance addiction and disorders
F14.23	316	Cocaine dependence with withdrawal	Substance addiction and disorders
F14.24	316	Cocaine dependence with cocaine-induced mood disorder	Substance addiction and disorders
F14.250	316	Cocaine dependence with cocaine-induced psychotic disorder with delusions	Substance addiction and disorders
F14.251	316	Cocaine dependence with cocaine-induced psychotic disorder with hallucinations	Substance addiction and disorders
F14.259	316	Cocaine dependence with cocaine-induced psychotic disorder, unspecified	Substance addiction and disorders
F14.280	316	Cocaine dependence with cocaine-induced anxiety disorder	Substance addiction and disorders
F14.282	316	Cocaine dependence with cocaine-induced sleep disorder	Substance addiction and disorders
F14.288	316	Cocaine dependence with other cocaine-induced disorder	Substance addiction and disorders
F14.29	316	Cocaine dependence with unspecified cocaine-induced disorder	Substance addiction and disorders
F15.10	316	Other stimulant abuse, uncomplicated	Substance addiction and disorders
F15.120	316	Other stimulant abuse with intoxication, uncomplicated	Substance addiction and disorders
F15.122	316	Other stimulant abuse with intoxication with perceptual disturbance	Substance addiction and disorders
F15.129	316	Other stimulant abuse with intoxication, unspecified	Substance addiction and disorders
F15.14	316	Other stimulant abuse with stimulant-induced mood disorder	Substance addiction and disorders
F15.150	316	Other stimulant abuse with stimulant-induced psychotic disorder with delusions	Substance addiction and disorders
F15.151	316	Other stimulant abuse with stimulant-induced psychotic disorder with hallucinations	Substance addiction and disorders
F15.159	316	Other stimulant abuse with stimulant-induced psychotic disorder, unspecified	Substance addiction and disorders
F15.180	316	Other stimulant abuse with stimulant-induced anxiety disorder	Substance addiction and disorders
F15.182	316	Other stimulant abuse with stimulant-induced sleep disorder	Substance addiction and disorders
F15.188	316	Other stimulant abuse with other stimulant-induced disorder	Substance addiction and disorders
F15.19	316	Other stimulant abuse with unspecified stimulant-induced disorder	Substance addiction and disorders
F15.20	316	Other stimulant dependence, uncomplicated	Substance addiction and disorders

© 2022 Barr PB et al. *JAMA Psychiatry*

F15.21	316	Other stimulant dependence, in remission	Substance addiction and disorders
F15.220	316	Other stimulant dependence with intoxication, uncomplicated	Substance addiction and disorders
F15.221	316	Other stimulant dependence with intoxication delirium	Substance addiction and disorders
F15.222	316	Other stimulant dependence with intoxication with perceptual disturbance	Substance addiction and disorders
F15.229	316	Other stimulant dependence with intoxication, unspecified	Substance addiction and disorders
F15.23	316	Other stimulant dependence with withdrawal	Substance addiction and disorders
F15.24	316	Other stimulant dependence with stimulant-induced mood disorder	Substance addiction and disorders
F15.250	316	Other stimulant dependence with stimulant-induced psychotic disorder with delusions	Substance addiction and disorders
F15.251	316	Other stimulant dependence with stimulant-induced psychotic disorder with hallucinations	Substance addiction and disorders
F15.259	316	Other stimulant dependence with stimulant-induced psychotic disorder, unspecified	Substance addiction and disorders
F15.280	316	Other stimulant dependence with stimulant-induced anxiety disorder	Substance addiction and disorders
F15.282	316	Other stimulant dependence with stimulant-induced sleep disorder	Substance addiction and disorders
F15.288	316	Other stimulant dependence with other stimulant-induced disorder	Substance addiction and disorders
F15.29	316	Other stimulant dependence with unspecified stimulant-induced disorder	Substance addiction and disorders
F16.1	316	Hallucinogen abuse	Substance addiction and disorders
F16.10	316	Hallucinogen abuse, uncomplicated	Substance addiction and disorders
F16.120	316	Hallucinogen abuse with intoxication, uncomplicated	Substance addiction and disorders
F16.122	316	Hallucinogen abuse with intoxication with perceptual disturbance	Substance addiction and disorders
F16.129	316	Hallucinogen abuse with intoxication, unspecified	Substance addiction and disorders
F16.14	316	Hallucinogen abuse with hallucinogen-induced mood disorder	Substance addiction and disorders
F16.151	316	Hallucinogen abuse with hallucinogen-induced psychotic disorder with hallucinations	Substance addiction and disorders
F16.183	316	Hallucinogen abuse with hallucinogen persisting perception disorder (flashbacks)	Substance addiction and disorders
F16.20	316	Hallucinogen dependence, uncomplicated	Substance addiction and disorders
F16.21	316	Hallucinogen dependence, in remission	Substance addiction and disorders
F16.229	316	Hallucinogen dependence with intoxication, unspecified	Substance addiction and disorders
F16.24	316	Hallucinogen dependence with hallucinogen-induced mood disorder	Substance addiction and disorders
F16.283	316	Hallucinogen dependence with hallucinogen persisting perception disorder (flashbacks)	Substance addiction and disorders
F16.288	316	Hallucinogen dependence with other hallucinogen-induced disorder	Substance addiction and disorders
F18.10	316	Inhalant abuse, uncomplicated	Substance addiction and disorders
F18.14	316	Inhalant abuse with inhalant-induced mood disorder	Substance addiction and disorders
F18.19	316	Inhalant abuse with unspecified inhalant-induced disorder	Substance addiction and disorders
F18.20	316	Inhalant dependence, uncomplicated	Substance addiction and disorders
F18.21	316	Inhalant dependence, in remission	Substance addiction and disorders
F18.29	316	Inhalant dependence with unspecified inhalant-induced disorder	Substance addiction and disorders
F19.10	316	Other psychoactive substance abuse, uncomplicated	Substance addiction and disorders
F19.120	316	Other psychoactive substance abuse with intoxication, uncomplicated	Substance addiction and disorders

© 2022 Barr PB et al. *JAMA Psychiatry*

F19.122	316	Other psychoactive substance abuse with intoxication with perceptual disturbances	Substance addiction and disorders
F19.129	316	Other psychoactive substance abuse with intoxication, unspecified	Substance addiction and disorders
F19.14	316	Other psychoactive substance abuse with psychoactive substance-induced mood disorder	Substance addiction and disorders
F19.150	316	Other psychoactive substance abuse with psychoactive substance-induced psychotic disorder with delusions	Substance addiction and disorders
F19.151	316	Other psychoactive substance abuse with psychoactive substance-induced psychotic disorder with hallucinations	Substance addiction and disorders
F19.159	316	Other psychoactive substance abuse with psychoactive substance-induced psychotic disorder, unspecified	Substance addiction and disorders
F19.180	316	Other psychoactive substance abuse with psychoactive substance-induced anxiety disorder	Substance addiction and disorders
F19.181	316	Other psychoactive substance abuse with psychoactive substance-induced sexual dysfunction	Substance addiction and disorders
F19.182	316	Other psychoactive substance abuse with psychoactive substance-induced sleep disorder	Substance addiction and disorders
F19.188	316	Other psychoactive substance abuse with other psychoactive substance-induced disorder	Substance addiction and disorders
F19.19	316	Other psychoactive substance abuse with unspecified psychoactive substance-induced disorder	Substance addiction and disorders
F19.20	316	Other psychoactive substance dependence, uncomplicated	Substance addiction and disorders
F19.21	316	Other psychoactive substance dependence, in remission	Substance addiction and disorders
F19.220	316	Other psychoactive substance dependence with intoxication, uncomplicated	Substance addiction and disorders
F19.221	316	Other psychoactive substance dependence with intoxication delirium	Substance addiction and disorders
F19.229	316	Other psychoactive substance dependence with intoxication, unspecified	Substance addiction and disorders
F19.230	316	Other psychoactive substance dependence with withdrawal, uncomplicated	Substance addiction and disorders
F19.231	316	Other psychoactive substance dependence with withdrawal delirium	Substance addiction and disorders
F19.232	316	Other psychoactive substance dependence with withdrawal with perceptual disturbance	Substance addiction and disorders
F19.239	316	Other psychoactive substance dependence with withdrawal, unspecified	Substance addiction and disorders
F19.24	316	Other psychoactive substance dependence with psychoactive substance-induced mood disorder	Substance addiction and disorders
F19.250	316	Other psychoactive substance dependence with psychoactive substance-induced psychotic disorder with delusions	Substance addiction and disorders
F19.251	316	Other psychoactive substance dependence with psychoactive substance-induced psychotic disorder with hallucinations	Substance addiction and disorders
F19.259	316	Other psychoactive substance dependence with psychoactive substance-induced psychotic disorder, unspecified	Substance addiction and disorders
F19.280	316	Other psychoactive substance dependence with psychoactive substance-induced anxiety disorder	Substance addiction and disorders
F19.288	316	Other psychoactive substance dependence with other psychoactive substance-induced disorder	Substance addiction and disorders
F19.29	316	Other psychoactive substance dependence with unspecified psychoactive substance-induced disorder	Substance addiction and disorders
F10.1	317	Alcohol abuse	Alcohol-related disorders
F10.10	317	Alcohol abuse, uncomplicated	Alcohol-related disorders
F10.120	317	Alcohol abuse with intoxication, uncomplicated	Alcohol-related disorders
F10.129	317	Alcohol abuse with intoxication, unspecified	Alcohol-related disorders
F10.14	317	Alcohol abuse with alcohol-induced mood disorder	Alcohol-related disorders
F10.150	317	Alcohol abuse with alcohol-induced psychotic disorder with delusions	Alcohol-related disorders
F10.151	317	Alcohol abuse with alcohol-induced psychotic disorder with hallucinations	Alcohol-related disorders
F10.159	317	Alcohol abuse with alcohol-induced psychotic disorder, unspecified	Alcohol-related disorders
F10.180	317	Alcohol abuse with alcohol-induced anxiety disorder	Alcohol-related disorders
F10.182	317	Alcohol abuse with alcohol-induced sleep disorder	Alcohol-related disorders

F10.188	317	Alcohol abuse with other alcohol-induced disorder	Alcohol-related disorders
F10.19	317	Alcohol abuse with unspecified alcohol-induced disorder	Alcohol-related disorders
F10.2	317	Alcohol dependence	Alcohol-related disorders
F10.20	317	Alcohol dependence, uncomplicated	Alcohol-related disorders
F10.21	317	Alcohol dependence, in remission	Alcohol-related disorders
F10.22	317	Alcohol dependence with intoxication	Alcohol-related disorders
F10.220	317	Alcohol dependence with intoxication, uncomplicated	Alcohol-related disorders
F10.229	317	Alcohol dependence with intoxication, unspecified	Alcohol-related disorders
F10.230	317	Alcohol dependence with withdrawal, uncomplicated	Alcohol-related disorders
F10.232	317	Alcohol dependence with withdrawal with perceptual disturbance	Alcohol-related disorders
F10.239	317	Alcohol dependence with withdrawal, unspecified	Alcohol-related disorders
F10.24	317	Alcohol dependence with alcohol-induced mood disorder	Alcohol-related disorders
F10.250	317	Alcohol dependence with alcohol-induced psychotic disorder with delusions	Alcohol-related disorders
F10.251	317	Alcohol dependence with alcohol-induced psychotic disorder with hallucinations	Alcohol-related disorders
F10.259	317	Alcohol dependence with alcohol-induced psychotic disorder, unspecified	Alcohol-related disorders
F10.26	317	Alcohol dependence with alcohol-induced persisting amnestic disorder	Alcohol-related disorders
F10.27	317	Alcohol dependence with alcohol-induced persisting dementia	Alcohol-related disorders
F10.280	317	Alcohol dependence with alcohol-induced anxiety disorder	Alcohol-related disorders
F10.282	317	Alcohol dependence with alcohol-induced sleep disorder	Alcohol-related disorders
F10.288	317	Alcohol dependence with other alcohol-induced disorder	Alcohol-related disorders
F10.29	317	Alcohol dependence with unspecified alcohol-induced disorder	Alcohol-related disorders
F10.1	317.1	Alcohol abuse	Alcoholism
F10.10	317.1	Alcohol abuse, uncomplicated	Alcoholism
F10.120	317.1	Alcohol abuse with intoxication, uncomplicated	Alcoholism
F10.129	317.1	Alcohol abuse with intoxication, unspecified	Alcoholism
F10.151	317.1	Alcohol abuse with alcohol-induced psychotic disorder with hallucinations	Alcoholism
F10.230	317.1	Alcohol dependence with withdrawal, uncomplicated	Alcoholism
F10.232	317.1	Alcohol dependence with withdrawal with perceptual disturbance	Alcoholism
F10.239	317.1	Alcohol dependence with withdrawal, unspecified	Alcoholism
F10.251	317.1	Alcohol dependence with alcohol-induced psychotic disorder with hallucinations	Alcoholism
F10.26	317.1	Alcohol dependence with alcohol-induced persisting amnestic disorder	Alcoholism
F10.27	317.1	Alcohol dependence with alcohol-induced persisting dementia	Alcoholism
F17.200	318	Nicotine dependence, unspecified, uncomplicated	Tobacco use disorder
F17.201	318	Nicotine dependence, unspecified, in remission	Tobacco use disorder
F17.210	318	Nicotine dependence, cigarettes, uncomplicated	Tobacco use disorder
F17.211	318	Nicotine dependence, cigarettes, in remission	Tobacco use disorder
F17.220	318	Nicotine dependence, chewing tobacco, uncomplicated	Tobacco use disorder
F17.221	318	Nicotine dependence, chewing tobacco, in remission	Tobacco use disorder
F17.290	318	Nicotine dependence, other tobacco product, uncomplicated	Tobacco use disorder

© 2022 Barr PB et al. *JAMA Psychiatry*

F17.291	318	Nicotine dependence, other tobacco product, in remission	Tobacco use disorder
F51.01	327	Primary insomnia	Sleep disorders
F51.02	327	Adjustment insomnia	Sleep disorders
F51.03	327	Paradoxical insomnia	Sleep disorders
F51.04	327	Psychophysiologic insomnia	Sleep disorders
F51.05	327	Insomnia due to other mental disorder	Sleep disorders
F51.09	327	Other insomnia not due to a substance or known physiological condition	Sleep disorders
F51.11	327	Primary hypersomnia	Sleep disorders
F51.12	327	Insufficient sleep syndrome	Sleep disorders
F51.13	327	Hypersomnia due to other mental disorder	Sleep disorders
F51.19	327	Other hypersomnia not due to a substance or known physiological condition	Sleep disorders
F51.3	327	Sleepwalking [somnambulism]	Sleep disorders
F51.4	327	Sleep terrors [night terrors]	Sleep disorders
F51.5	327	Nightmare disorder	Sleep disorders
F51.8	327	Other sleep disorders not due to a substance or known physiological condition	Sleep disorders
F51.9	327	Sleep disorder not due to a substance or known physiological condition, unspecified	Sleep disorders
F51.11	327.1	Primary hypersomnia	Hypersomnia
F51.12	327.1	Insufficient sleep syndrome	Hypersomnia
F51.13	327.1	Hypersomnia due to other mental disorder	Hypersomnia
F51.19	327.1	Other hypersomnia not due to a substance or known physiological condition	Hypersomnia
F51.01	327.4	Primary insomnia	Insomnia
F51.02	327.4	Adjustment insomnia	Insomnia
F51.03	327.4	Paradoxical insomnia	Insomnia
F51.04	327.4	Psychophysiologic insomnia	Insomnia
F51.05	327.4	Insomnia due to other mental disorder	Insomnia
F51.09	327.4	Other insomnia not due to a substance or known physiological condition	Insomnia
F51.01	327.41	Primary insomnia	Organic or persistent insomnia
F51.03	327.41	Paradoxical insomnia	Organic or persistent insomnia
F51.09	327.41	Other insomnia not due to a substance or known physiological condition	Organic or persistent insomnia
F51.3	327.5	Sleepwalking [somnambulism]	Parasomnia
F51.4	327.5	Sleep terrors [night terrors]	Parasomnia
F51.5	327.5	Nightmare disorder	Parasomnia

© 2022 Barr PB et al. *JAMA Psychiatry*

7 ETABLE 2: ALL OF US SAMPLE CHARACTERISTICS (N = 329,038)

	N	%
Sex assigned at birth: Female	199,658	60.68%
Sex assigned at birth Male	125,184	38.05%
Sex assigned at birth Not male, not female, no matching concept, prefer not to answer, or skipped	4,196	1.28%
Gender: Woman	197,961	60.16%
Gender: Man	124,202	37.75%
Gender: Not man only, not woman only, no matching concept, prefer not to answer, or skipped	6,875	2.09%
Sexual orientation: Straight/Heterosexual	290,176	88.19%
Sexual orientation: All others	38,862	11.81%
Age: 18-29	48,438	14.72%
Age: 30-44	74,353	22.60%
Age: 45-64	128,463	39.04%
Age: 65+	77,784	23.64%
Race-ethnicity: Non-Hispanic White	172,753	52.50%
Race-ethnicity: Black/African American	69,084	21.00%
Race-ethnicity: Hispanic or Latino/a/x	59,348	18.04%
Race-ethnicity: Asian	10,747	3.27%
Race-ethnicity: Other race-ethnicity	5,697	1.73%
Race-ethnicity: Multiracial	5,282	1.61%
Race-ethnicity: Missing	6,127	1.86%
Education: Less than high school	32,178	9.78%
Education: High school or equivalent	65,214	19.82%
Education: Some college	83,518	25.38%
Education: College grad/advanced degree	140,542	42.71%
Education: Missing	7,586	2.31%
Household Income: Less than 25k	90,190	27.41%
Household Income:25k to 50k	49,190	14.95%
Household Income:50k to 75k	33,696	10.24%
Household Income:75k 100k	25,867	7.86%
Household Income: more than 100k	66,389	20.18%
Household Income: Missing	63,706	19.36%
Health insurance: Yes	296,627	90.15%
Health insurance: No	23,235	7.06%
Health insurance: Missing	9,176	2.79%
Marital status: Married	137,597	41.82%
Marital status: Living With Partner	21,906	6.66%
Marital status: Divorced	46,496	14.13%
Marital status: Never Married	85,479	25.98%
Marital status: Separated	11,655	3.54%
Marital status: Widowed	16,779	5.10%
Marital status: Missing	9,126	2.77%
Birthplace: USA	275,464	83.72%
Birthplace: Foreign born	50,237	15.27%
Birthplace: Missing	3,337	1.01%

© 2022 Barr PB et al. *JAMA Psychiatry*

8 ETABLE 3: PREVALENCE ACROSS ICD10CM CODE THRESHOLDS

	1+ ICD codes		2+ ICD codes		3+ ICD codes		4+ ICD codes	
	N	Prev.	*N*	Prev.	*N*	Prev.	*N*	Prev.
Schizophrenia	3,562	1.08%	2,653	0.81%	2,218	0.67%	1,941	0.59%
Bipolar disorder	10,577	3.21%	7,411	2.25%	5,950	1.81%	5,085	1.55%
Major depressive disorder	41,493	12.61%	30,544	9.28%	24,695	7.51%	20,770	6.31%
Adjustment disorder	8,565	2.60%	4,565	1.39%	2,985	0.91%	2,227	0.68%
Anxiety disorder (unspecified)	42,309	12.86%	29,989	9.11%	23,669	7.19%	19,677	5.98%
Alcohol use disorder	9,725	2.96%	6,201	1.88%	4,678	1.42%	3,821	1.16%
Nicotine use disorder	25,515	7.75%	17,272	5.25%	13,169	4.00%	10,529	3.20%
Dysthymic disorder	2,924	0.89%	1,661	0.50%	1,201	0.37%	971	0.30%
Generalized anxiety disorder	12,736	3.87%	8,320	2.53%	6,467	1.97%	5,341	1.62%
Post-traumatic stress disorder	7,643	2.32%	5,525	1.68%	4,532	1.38%	3,929	1.19%
Attention-deficit hyperactivity disorder	3,588	1.09%	2,475	0.75%	1,950	0.59%	1,619	0.49%
Social anxiety disorder/agoraphobia	5,365	1.63%	2,934	0.89%	2,057	0.63%	1,572	0.48%
Eating disorder	1,014	0.31%	559	0.17%	403	0.12%	310	0.09%
Phobia	1,236	0.38%	406	0.12%	195	0.06%	123	0.04%
Sleep disorder	6,591	2.00%	3,511	1.07%	2,338	0.71%	1,735	0.53%
Drug use disorder	14,301	4.35%	9,907	3.01%	7,692	2.34%	6,307	1.92%
Antisocial/borderline personality disorder	1,396	0.42%	894	0.27%	675	0.21%	571	0.17%
Other personality disorder	2,327	0.71%	1,497	0.45%	1,150	0.35%	979	0.30%
Obsessive compulsive disorder	942	0.29%	603	0.18%	464	0.14%	383	0.12%
Anorexia nervosa	148	0.04%	85	0.03%	61	0.02%	54	0.02%
Schizotypal personality disorder	83	0.03%	45	0.01%	41	0.01%	35	0.01%
Total number of cases (not mutually exclusive)	202,040		202,040		202,040		87,979	

N = frequency count; Prev. = prevalence

© 2022 Barr PB et al. *JAMA Psychiatry*

9 ETABLE 4: ADJUSTED ESTIMATES OF RISK FOR PSYCHIATRIC DISORDERS (3+ ICD CODES)

	MOOD			ANX			SUD			STRESS			SCZ			PERS		
	OR	SE		OR	SE		OR	SE		OR	SE		OR	SE		OR	SE	
Female (ref)	-	-		-	-		-	-		-	-		-	-		-	-	
Male	0.83	0.07		0.80	0.08	*	1.29	0.08	*	0.85	0.13		0.91	0.22		0.67	0.28	
Neither male nor female selected	1.02	0.06		1.01	0.07		1.25	0.07	*	1.00	0.11		0.90	0.21		0.58	0.33	
Woman (ref)	-	-		-	-		-	-		-	-		-	-		-	-	
Man	0.83	0.07	*	0.81	0.08	*	1.25	0.08	*	1.12	0.13		2.01	0.22	*	1.02	0.28	
Neither man nor women selected	0.99	0.05		0.95	0.05		1.11	0.06		1.27	0.09	*	1.62	0.17	*	1.53	0.18	
Straight/Heterosexual	-	-		-	-		-	-		-	-		-	-		-	-	
Other than straight/heterosexual	1.37	0.02	*	1.25	0.02	*	1.19	0.02	*	1.44	0.03	*	1.55	0.06	*	1.87	0.07	*
Age 18-29 (ref)	-	-		-	-		-	-		-	-		-	-		-	-	
Age 30-44	1.68	0.02	*	1.57	0.02	*	2.36	0.03	*	1.65	0.05	*	2.52	0.08	*	1.30	0.09	*
Age 45-64	2.05	0.02	*	1.56	0.02	*	2.58	0.03	*	1.73	0.04	*	2.68	0.08	*	1.09	0.09	
Age 65 and older	1.59	0.03	*	1.09	0.03	*	1.29	0.04	*	1.19	0.05	*	1.14	0.11		0.42	0.13	*
Non-Hispanic White (ref)	-	-		-	-		-	-		-	-		-	-		-	-	
Black/African American	0.51	0.02	*	0.39	0.02	*	0.66	0.02	*	0.63	0.04	*	1.26	0.06	*	0.32	0.09	*
Hispanic/Latino/a/x	0.72	0.02	*	0.65	0.02	*	0.72	0.03	*	0.73	0.04	*	1.13	0.08		0.42	0.11	*
Asian	0.46	0.06	*	0.44	0.06	*	0.40	0.11	*	0.58	0.11	*	0.43	0.36		0.31	0.34	*
Other race-ethnicity	0.94	0.05		0.83	0.05	*	1.11	0.06		0.99	0.09		1.39	0.16		0.80	0.20	
Multiracial	0.87	0.05	*	0.80	0.05	*	0.98	0.06		1.06	0.09		1.84	0.15	*	0.83	0.20	
No race-ethnicity listed	0.84	0.04	*	0.78	0.05	*	0.96	0.05		0.86	0.09		1.53	0.13	*	0.60	0.21	
US born (ref)	-	-		-	-		-	-		-	-		-	-		-	-	
Foreign born	0.66	0.02	*	0.60	0.03	*	0.35	0.04	*	0.62	0.05	*	0.46	0.10	*	0.55	0.14	*
No birthplace reported	0.95	0.06		0.91	0.07		1.09	0.06		0.84	0.12		1.09	0.16		0.84	0.29	
Less than HS (ref)	-	-		-	-		-	-		-	-		-	-		-	-	
HS diploma or equivalent	0.95	0.02		0.98	0.03		0.83	0.02	*	1.04	0.04		0.77	0.06	*	0.88	0.10	
Some college	0.95	0.02		0.99	0.03		0.66	0.03	*	1.06	0.05		0.58	0.07	*	1.02	0.10	
College degree or higher	0.68	0.02	*	0.72	0.03	*	0.27	0.03	*	0.78	0.05	*	0.36	0.09	*	0.54	0.12	*
No education reported	0.89	0.05		0.84	0.05	*	0.92	0.05		0.90	0.09		0.82	0.11		0.90	0.21	
Income less than 25K (ref)	-	-		-	-		-	-		-	-		-	-		-	-	
Income 25-50K	0.67	0.02	*	0.74	0.02	*	0.54	0.03	*	0.67	0.04	*	0.36	0.09	*	0.52	0.09	*
Income 50-75K	0.54	0.02	*	0.61	0.03	*	0.33	0.04	*	0.59	0.05	*	0.17	0.16	*	0.37	0.13	*
Income 75-100K	0.43	0.03	*	0.53	0.03	*	0.26	0.05	*	0.47	0.06	*	0.10	0.27	*	0.27	0.18	*
Income 100K or more	0.31	0.03	*	0.39	0.03	*	0.16	0.05	*	0.34	0.05	*	0.04	0.28	*	0.11	0.19	*
No income reported	0.66	0.02	*	0.72	0.02	*	0.71	0.02	*	0.68	0.04	*	0.73	0.05	*	0.67	0.08	*

© 2022 Barr PB et al. *JAMA Psychiatry*

Have health insurance (ref)	-	-		-	-		-	-		-	-		-	-		-	-	
No health insurance	0.41	0.03	*	0.41	0.03	*	0.38	0.03	*	0.58	0.06	*	0.29	0.10	*	0.59	0.13	*
No health insurance info reported	0.67	0.04	*	0.69	0.05	*	0.74	0.04	*	0.73	0.08	*	0.69	0.11	*	0.59	0.21	
Married (ref)	1.12	0.03	*	1.10	0.03	*	1.84	0.04	*	1.12	0.05		1.65	0.11	*	1.27	0.14	
Cohabiting	1.42	0.02	*	1.28	0.02	*	1.95	0.03	*	1.52	0.04	*	2.13	0.09	*	2.20	0.10	*
Divorced	1.24	0.02	*	1.10	0.02	*	1.76	0.03	*	1.16	0.04	*	2.49	0.08	*	1.67	0.10	*
Never married	1.40	0.03	*	1.20	0.04	*	1.98	0.04	*	1.44	0.06	*	2.08	0.11	*	1.91	0.15	*
Separated	1.33	0.03	*	1.16	0.03	*	1.78	0.04	*	1.36	0.06	*	1.98	0.12	*	1.42	0.17	
Widowed	1.20	0.04	*	1.16	0.04	*	1.82	0.05	*	1.37	0.07	*	2.49	0.12	*	2.36	0.16	*
No marital info reported	1.12	0.03	*	1.10	0.03	*	1.84	0.04	*	1.12	0.05		1.65	0.11	*	1.27	0.14	

* *p* < .05/6 = .0083
OR = odds ratio; SE = standard error; MOOD = any mood disorder; ANX = any anxiety disorder; SUD = any substance use disorder; STRESS = any stress-related disorder; SCZ = schizophrenia; PERS = any personality disorder. All estimates conditional on all other covariates included in the model.

© 2022 Barr PB et al. *JAMA Psychiatry*

10 ETABLE 5: ADJUSTED ESTIMATES OF RISK FOR PSYCHIATRIC DISORDERS (4+ ICD CODES)

	MOOD			ANX			SUD			STRESS			SCZ			PERS		
	OR	SE		OR	SE		OR	SE		OR	SE		OR	SE		OR	SE	
Female (ref)	-	-		-	-		-	-		-	-		-	-		-	-	
Male	0.87	0.08		0.80	0.08	*	1.28	0.09	*	0.88	0.14		1.06	0.23		0.58	0.30	
Neither male nor female selected	1.03	0.06		1.00	0.07		1.21	0.08		1.00	0.13		1.16	0.22		0.38	0.38	
Woman (ref)	-	-		-	-		-	-		-	-		-	-		-	-	
Man	0.80	0.08	*	0.81	0.08		1.23	0.09		1.13	0.14		1.76	0.23		1.17	0.31	
Neither man nor women selected	0.97	0.05		0.95	0.06		1.15	0.07		1.31	0.10	*	1.33	0.19		1.74	0.19	*
Straight/Heterosexual	-	-		-	-		-	-		-	-		-	-		-	-	
Other than straight/heterosexual	1.37	0.02	*	1.27	0.02	*	1.20	0.02	*	1.46	0.04	*	1.57	0.06	*	1.92	0.08	*
Age 18-29 (ref)	-	-		-	-		-	-		-	-		-	-		-	-	
Age 30-44	1.69	0.03	*	1.58	0.03	*	2.43	0.03	*	1.67	0.05	*	2.61	0.09	*	1.34	0.10	*
Age 45-64	2.11	0.02	*	1.58	0.03	*	2.72	0.03	*	1.78	0.05	*	2.79	0.09	*	1.14	0.10	
Age 65 and older	1.62	0.03	*	1.11	0.03	*	1.33	0.04	*	1.22	0.06	*	1.24	0.12		0.44	0.15	*
Non-Hispanic White (ref)	-	-		-	-		-	-		-	-		-	-		-	-	
Black/African American	0.51	0.02	*	0.39	0.02	*	0.65	0.02	*	0.62	0.04	*	1.21	0.06	*	0.31	0.10	*
Hispanic/Latino/a/x	0.71	0.02	*	0.64	0.02	*	0.68	0.03	*	0.71	0.05	*	1.08	0.08		0.42	0.12	*
Asian	0.45	0.06	*	0.41	0.07	*	0.38	0.12	*	0.58	0.12	*	0.48	0.36		0.28	0.39	*
Other race-ethnicity	0.94	0.05		0.82	0.05	*	1.09	0.06		0.95	0.10		1.34	0.17		0.56	0.26	
Multiracial	0.85	0.05	*	0.81	0.05	*	0.97	0.07		1.11	0.10		1.80	0.16	*	0.78	0.22	
No race-ethnicity listed	0.84	0.05	*	0.76	0.05	*	1.00	0.06		0.82	0.10		1.51	0.14	*	0.61	0.23	
US born (ref)	-	-		-	-		-	-		-	-		-	-		-	-	
Foreign born	0.66	0.03	*	0.60	0.03	*	0.35	0.04	*	0.60	0.06	*	0.47	0.11	*	0.57	0.15	*
No birthplace reported	0.99	0.06		0.91	0.07		1.09	0.07		0.82	0.13		1.18	0.17		0.96	0.30	
Less than HS (ref)	-	-		-	-		-	-		-	-		-	-		-	-	
HS diploma or equivalent	0.96	0.02		1.01	0.03		0.82	0.03	*	1.03	0.05		0.77	0.06	*	0.90	0.12	
Some college	0.96	0.02		1.02	0.03		0.66	0.03	*	1.08	0.05		0.57	0.07	*	1.09	0.12	
College degree or higher	0.68	0.03	*	0.73	0.03	*	0.27	0.04	*	0.78	0.05	*	0.37	0.10	*	0.60	0.13	*
No education reported	0.89	0.05		0.89	0.06		0.91	0.05		0.87	0.10		0.75	0.12		0.97	0.23	
	0.96	0.02		1.01	0.03		0.82	0.03	*	1.03	0.05		0.77	0.06	*	0.90	0.12	
Income less than 25K (ref)	-	-		-	-		-	-		-	-		-	-		-	-	
Income 25-50K	0.66	0.02	*	0.73	0.02	*	0.52	0.03	*	0.66	0.04	*	0.36	0.09	*	0.53	0.10	*
Income 50-75K	0.53	0.03	*	0.61	0.03	*	0.33	0.04	*	0.62	0.05	*	0.16	0.18	*	0.37	0.14	*
Income 75-100K	0.42	0.03	*	0.52	0.03	*	0.25	0.06	*	0.46	0.07	*	0.09	0.29	*	0.26	0.19	*
Income 100K or more	0.30	0.03	*	0.39	0.03	*	0.15	0.05	*	0.34	0.06	*	0.04	0.29	*	0.11	0.21	*
No income reported	0.64	0.02	*	0.71	0.02	*	0.71	0.02	*	0.67	0.04	*	0.69	0.06	*	0.66	0.09	*

© 2022 Barr PB et al. *JAMA Psychiatry*

Have health insurance (ref)	-	-		-	-		-	-		-	-		-	-		-	-	
No health insurance	0.41	0.03	*	0.43	0.04	*	0.36	0.04	*	0.59	0.06	*	0.30	0.11	*	0.58	0.14	*
No health insurance info reported	0.66	0.05	*	0.70	0.05	*	0.69	0.05	*	0.75	0.09	*	0.71	0.12	*	0.56	0.23	
Married (ref)	1.14	0.03	*	1.10	0.03	*	1.86	0.04	*	1.17	0.06		1.64	0.12	*	1.15	0.15	
Cohabiting	1.43	0.02	*	1.30	0.02	*	1.99	0.03	*	1.58	0.04	*	2.21	0.09	*	2.27	0.11	*
Divorced	1.24	0.02	*	1.11	0.02	*	1.83	0.03	*	1.20	0.04	*	2.54	0.09	*	1.70	0.11	*
Never married	1.43	0.03	*	1.21	0.04	*	2.07	0.04	*	1.53	0.07	*	2.12	0.12	*	2.19	0.16	*
Separated	1.33	0.03	*	1.14	0.03	*	1.79	0.04	*	1.34	0.06	*	2.15	0.13	*	1.29	0.19	
Widowed	1.20	0.04	*	1.17	0.05	*	1.90	0.05	*	1.46	0.08	*	2.55	0.13	*	2.35	0.18	*
No marital info reported	1.14	0.03	*	1.10	0.03	*	1.86	0.04	*	1.17	0.06		1.64	0.12	*	1.15	0.15	

* $p < .05/6 = .0083$
OR = odds ratio; SE = standard error; MOOD = any mood disorder; ANX = any anxiety disorder; SUD = any substance use disorder; STRESS = any stress-related disorder; SCZ = schizophrenia; PERS = any personality disorder. All estimates conditional on all other covariates included in the model.

© 2022 Barr PB et al. *JAMA Psychiatry*
